# Activated microglial phenotypes in the hippocampal CA2 subfield are implicated in Lewy body disease progression

**DOI:** 10.1007/s00401-026-03065-8

**Published:** 2026-08-08

**Authors:** Esteban Luna, Katheryn A. Q. Cousins, Sheina Emrani, Sharon X. Xie, Winifred Trotman, Noah Capp, Philip Sabatini, Parham Pouladvand, EunRan Suh, Vivianna M. Van Deerlin, Daniel Weintraub, Alice S. Chen-Plotkin, Edward B. Lee, David J. Irwin

**Affiliations:** 1 Department of Neurology, Perelman School of Medicine at the University of Pennsylvania, Philadelphia, PA 19104 USA; 2 Department of Biostatistics, Epidemiology and Informatics, Perelman School of Medicine at the University of Pennsylvania, Philadelphia, PA 19104 USA; 3 Department of Pathology and Laboratory Medicine, Center for Neurodegenerative Disease Research, Perelman School of Medicine at the University of Pennsylvania, Philadelphia, PA 19104 USA; 4 Department of Psychiatry, Perelman School of Medicine at the University of Pennsylvania, Philadelphia, PA 19104 USA

**Keywords:** Lewy body disease, Lewy body dementia, Microglia, Hippocampus, α-Synuclein, Lewy pathology

## Abstract

Histopathologic staging models of neuronal α-synuclein pathology (n-asyn) in Lewy body disease (LBD) seldom evaluate brain regions with direct synaptic connectivity to model the role of microglial processes. We address this gap by testing the hypothesis that, within the well-defined synaptic connectivity of the intrahippocampal circuit, n-asyn is associated with activated microglial phenotypes. We selected a cohort of autopsy-confirmed LBD patients and minimal age-related copathologies (*n* = 62) and a control cohort of cognitively healthy patients with isolated hippocampal tau accumulation (i.e., primary age-related tauopathy, PART; *n* = 12), to control for neurodegenerative pathology without amyloid plaques. We immunostained consecutive hippocampal sections for n-asyn and established markers of activated microglial phenotypes, Iba1, HLA-DR, and CD68. With validated digital histology methods, we measured percent area occupied (%AO) of each marker in 6 hippocampal subfields to compare and correlate microglial morphologic and proteomic activation phenotypes between cohorts and used linear mixed effects models to compare the %AO between subfields while covariying for demographics. We also constructed groups of n-asyn restricted to the cornu ammonis (CA) 2–3 subfields (Focal Subtype) or widespread n-asyn within additional subfields (Widespread Subtype) to model hypothesized n-asyn spread within the intrahippocampal circuit. LBD patients exhibited increased HLA-DR and CD68%AO in most hippocampal subfields compared with PART. In LBD patients, all microglial markers were the highest in the CA2. CA2 n-asyn correlated with HLA-DR and CD68 but not Iba1%AO. Patients classified as Widespread Subtype had worse cognitive impairment and increased CA2 HLA-DR and CD68%AO. CA2 HLA-DR and CD68%AO correlated with distal n-asyn in retrograde, but not anterograde connected subfields. Our data show that activated microglial phenotypes in the CA2 of LBD patients are associated with worse clinical outcomes and retrograde n-asyn transmission. These data suggest that measures of microglial states can refine LBD histopathological progression models.

**Supplementary Information:**

The online version contains supplementary material available at 10.1007/s00401-026-03065-8.

## Introduction

Intraneuronal protein aggregates predominantly made up of α-synuclein, termed neuronal α-synuclein pathology (n-asyn), pathologically define Lewy body diseases (LBDs) that clinically encompass Parkinson’s disease (PD) without and with dementia (PDD) and dementia with Lewy bodies [34, 74, 76]. Braak and colleagues initially proposed a topographical pattern of n-asyn split into progressive stages for PD from the olfactory bulb and dorsal medulla with hypothesized spreading rostrally from the brainstem to limbic and neocortical regions [12, 13]. Subsequent postmortem staging systems define additional patterns of progressive n-asyn transsynaptic spread in the human brain and peripheral nervous system, but thus far, these schemata do not account for non-neuronal aspects of disease pathophysiology or provide detailed analysis within the anatomic framework of local circuits to model these intercellular interactions [2–4, 6, 50, 57, 61]. Indeed, genome-wide association studies in LBD have found risk variants in microglia-associated genes and genes with relative enriched expression in microglia over neurons [15, 65, 80]. Moreover, postmortem studies have found significant morphologic, transcriptional, and molecular changes in microglia within specific disease-relevant brain regions, such as the Substantia nigra pars compacta, suggesting a microglial component to disease pathogenesis [60, 75, 80]. Limited existing studies find a relatively modest increase in activated microglial phenotypes in LBD compared to the microglial response in Alzheimer’s disease (AD), suggesting an altered microglial response in LBD vs. AD [7, 23, 37, 38, 55, 59]. There is an established link between the development of activated microglial phenotypes and n-asyn within key vulnerable brain regions like the substantia nigra pars compacta, where n-asyn burden is associated with activated microglial states typified by increased phagocytic activity and antigen presentation through an increase in CD68 and HLA-DR, respectively [17, 23, 37, 38]. Despite this, the relationship between LBD clinical progression and these activated microglial phenotypes within the key vulnerable non-motor brain regions that develop n-asyn is unclear. The hippocampus provides an ideal anatomic model to test the association of activated microglial phenotypes that occur in response to progressive n-asyn burden in LBD because the subfields form part of the well-defined intra-hippocampal circuit that is critical to memory encoding and affected in LBD [39, 53]. The hippocampus develops n-asyn in dementia with Lewy bodies and in stage V of classical Braak staging of PD/PDD [1, 13]. The cornu ammonis 2 (CA2) hippocampal subfield is particularly vulnerable to forming n-asyn compared to other hippocampal subfields in LBD [1, 18, 21]. One study found that the CA2 develops increasing microglial phagocytic activity with increasing Parkinson’s Braak stage by measuring staining to CD68, and another found increased antigen presentation measured by HLA-DR in the CA2 of PD patients compared to controls [23, 38]. Despite this, detailed anatomic studies to model the relationship of activated microglial phenotypes with subregional n-asyn burden in the hippocampus are limited, and this is a crucial knowledge gap that could provide critical clues in elucidating the role of microglia in LBD pathogenesis.

Here, we tested the hypothesis that activated microglial phenotypes are associated with n-asyn burden within the hippocampal subfields in LBD. To do this, we selected a unique cohort of LBD patients with n-asyn and limited potentially confounding Alzheimer’s Disease Neuropathologic Change (ADNC) [30, 44]. We compared this select well-defined LBD cohort to a well-characterized control cohort of healthy adults diagnosed post-mortem with primary age-related tauopathy (PART), defined as limited age-related tau pathology within the CA2 with minimal amyloid plaques and carefully accounted for other age-related pathologies in our models [19, 85]. We used rigorously validated digital histopathology methods to measure the percent area occupied (%AO) of n-asyn and markers of activated microglial phenotypes including total microglial area, phagocytic activity, and antigen presentation with Iba1, CD68, and HLA-DR, respectively [18, 41, 49, 84, 89]. We found that LBD patients exhibit greater CD68 and HLA-DR signal in almost all hippocampal sub-regions compared to PART controls, despite only the CA2 and subiculum sub-regions showing a slightly increased Iba1%AO, suggestive of an enrichment of activated microglial phenotypes due to n-asyn. Moreover, we found that LBD patients with widespread hippocampal n-asyn throughout the hippocampal subfields demonstrate increased activated microglial phenotypes specifically within the CA2 and worse cognition compared to those with limited hippocampal n-asyn in the CA2–3 subfields alone. Activated microglial phenotypes in the CA2 correlated with n-asyn burden in distal subfields with retrograde connectivity to CA2–3 region but not anterograde activity within the intra-hippocampal circuit. Thus, contrary to our hypothesis, the data presented here implicate activated microglial phenotypes localized to the CA2 subfield in response to n-asyn in LBD in both advancing LBD clinical progression and neuropathological progression within the hippocampus. Furthermore, these data provide evidence that deeper phenotyping beyond n-asyn localization alone can further refine neuropathologic staging of LBD subtypes.

## Materials and methods

### Patient selection and study workflow

We selected patient samples from the Penn Integrated Neurodegenerative Disease Database (INDD) on 09/26/22 with the following criteria: To maximize the number of samples with n-asyn within the hippocampus, we first had primary inclusion criteria of a clinical diagnosis of PD, PDD, or dementia with Lewy bodies and primary neuropathologic diagnosis of LBD (i.e., brainstem, limbic or neocortical stage, *n* = 351). To reduce the confounds of mixed pathology, we excluded cases that had a clinically relevant medium- or high-level ADNC (*n* = 154 met inclusion criteria) [64]. We selected available tissue from the hippocampus at the level of the lateral geniculate nucleus for optimal sampling of all the 6 hippocampal subfields (CA 1–4, dentate gyrus [DG], subiculum [Sub]). Thus, we excluded cases with missing/damaged tissues and/or suboptimal sampling of the hippocampal subfields at this coronal level, resulting in a final cohort of 62 patients (Supplemental Fig. 1a). We included a control group of healthy adult brain donors without clinical neurologic disease, with a primary neuropathologic diagnosis of PART, minimal other age-related pathology, and available hippocampal tissue as above (*n* = 12).

### Neuropathologic assessment

Diagnostic neuropathologic assessment was performed as previously described using current neuropathological criteria for AD and LBD [64, 79]. Briefly, fresh brains obtained at the time of autopsy were fixed in either neutral buffered formalin or 70% ethanol overnight. The brain tissue was processed and embedded in paraffin as previously described. 6 μm thick sections were stained by immunohistochemistry (IHC) with validated antibodies to phosphorylated pathologic tau (pTau), amyloid-β, n-asyn, and TDP-43 [79]. *APOE* and *GBA* genotyping was performed as described and genetic data were obtained through the Integrated Neurodegenerative Disease Database [40, 88]. Ordinal rating scores for n-asyn using SYN303 obtained at autopsy in the CA region (CA1-4) of the hippocampus were obtained from INDD. All protocols were performed with informed consent and compliant with the University of Pennsylvania Institutional Review Board (IRB).

### Digital neuropathologic assessment

Near-adjacent tissue sections were stained by IHC in the Penn Digital Neuropathology Lab for phosphorylated-α-synuclein S129 as a marker of n-asyn (Abcam, MJF-R13, ab209421, 1:20k), Iba1 (FUJIFILM Wako, 019-19741, 1:4k, antigen retrieval with citric acid for 15 min), CD68 (Dako, M081401, 1:100, antigen retrieval with citric acid for 15 min), and HLA-DR (Genetex, MSVA-478R, GTX04457, 1:100, antigen retrieval with citric acid for 15 min) as previously described [31, 45]. Stained IHC sections were digitally scanned (Aperio AT2, Leica Biosystem, Wetzlar, Germany) at 20× magnification. Hippocampal subfield annotation and digital histology analysis were performed using QuPath Software (version 0.5.1) as previously described using previously validated cytoarchitectonic definitions of subfield delineations to generate a %AO of immunostain signal for each antibody in each region of interest (ROI; CA1-4, DG, Sub) from each whole-slide image of a unilateral hippocampal section for each patient that met all inclusion criteria [5, 18]. Briefly, the DG was identified by its characteristic densely packed granule cell layer. CA4 (hilus) was defined as the region enclosed by the blades of the DG. CA3 was identified by its large pyramidal neurons and proximity to the CA4, whereas CA2 was distinguished by a more compact pyramidal cell layer composed of relatively large pyramidal neurons located between CA3 and CA1. CA1 was identified by its broader pyramidal cell layer and transition to the Sub, which was recognized by its dispersed pyramidal neurons compared to the CA1 [18]. An illustrative example of hippocampal subfield annotation to measure CA1-4, DG, and Sub is shown (Supplemental Fig. 1b). Example signal thresholding to determine %AO of each stained marker is also shown (Supplemental Fig. 2).

### Clinical assessment

Clinical assessment of patients was done as part of ongoing observational research studies at Penn (P01-AG084497, P30-AG072979). These clinical assessments have been previously described [87]. Due to changes in batteries over the time period of autopsies, we focused on the Montreal Cognitive Assessment (MOCA) and Dementia Rating Scale-2 (DRS), which had the greatest amount of harmonized data and are relevant scores to memory. For patients with longitudinal data, the assessment closest to death was used to relate to postmortem data.

### Microglial morphologic analysis

The Iba1 IHC slides were quantified utilizing an automatic image processing pipeline. This method utilizes traditional color deconvolution techniques to extract the Iba1 DAB intensity from the RGB image [32]. Then we applied a unimodal thresholding algorithm to the histogram of DAB intensities to detect positive Iba1 reactivity [72]. Then, the microglia somas are detected via a method devised by Kayasandik et al., which first generates a soma probability map using anisotropic Gaussian filters, then segments this map using a fast march algorithm [43]. Each soma and attached microglial processes are analyzed by extracting various morphology features proposed by Leyh et al. [52]. Specific measurements acquired were soma area, hull area (measurement of total area encompassing soma and attached microglial processes), and critical radius (measurement of distance from soma to maximum number of process intersections) to capture morphological changes to Iba1-reactive microglia associated with activation phenotypes, where activated microglial phenotypes typically form larger soma area, reduced convex hull area and either increased or decreased critical radius [71].

### Neuronal counts

Brightfield images were color deconvolved in QuPath using the hematoxylin–DAB stain vector. For assessment of tissue morphology and nuclear architecture, only the hematoxylin channel was displayed and neuronal nuclei were manually counted by a single-blinded rater (EL) within the annotated CA2 subfield of CD68-stained slides.

### n-asyn hippocampal staging

To model progressive n-asyn spread through the intra-hippocampal circuit, we dichotomized patients into 2 groups or stages based on visual inspection. The group, termed Focal Subtype, was identified by n-asyn being relatively localized to the CA2–3 region with no or minimal n-asyn in other subfields. The group, termed Widespread Subtype, was identified as sections with substantial n-asyn distributed in hippocampal subfields beyond the CA2–3 region. Dichotomization was done by a single-blinded rater using solely the n-asyn-stained slides for grouping. There were no samples with n-asyn present predominantly outside of the CA2–3 region in this LBD cohort.

### Multiplex immunofluorescence

Multiplex immunofluorescence was performed on hippocampal tissue of n = 3 each of PART, Focal LBD and Widespread LBD Subtype samples on the Akoya PhenoCycler platform (Akoya Biosciences). To minimize confounds of amyloid copathology, these cases had negligible amyloid plaques. Sections were stained with fluorophore-bound oligonucleotide-conjugated antibodies against Iba1 (Genetex, GTX635363, barcode number BX033, 750nm reporter fluorophore), HLA-DR (Genetex, GTX04457, barcode number BX031, 647nm reporter fluorophore), CD68 (Abcam, ab303566, barcode number BX037, 750nm reporter fluorophore), n-asyn (MJFR13, Abcam, 209,421, barcode number BX027, 647 nm reporter fluorophore), pTau (AT8, Invitrogen, MN1020, barcode number BX025, 550nm reporter fluorophore), Amyloid Beta (NAB228, Invitrogen, 37-4200, barcode number BX024, 647 nm reporter fluorophore), NeuN (Abcam, ab209898, barcode number BX015, 647 reporter fluorophore), MAP2 (Abcam, ab236033, barcode number BX022, 750nm reporter fluorophore), and DAPI to characterize microglial phenotypes and the cellular colocalization of n-asyn. Briefly, slides were hydrated, antigen retrieval performed with 20 min of pressure cooker at 115 °C, followed by incubation with a cocktail of above primary antibodies in blocking buffer overnight at 4 °C. Next, fixation was performed with 1.6% paraformaldehyde in blocking buffer for 10 min at room temperature, slides incubated in methanol at 4 °C for 5 min to quench autofluorescence, followed by fusion reaction performed with reporter fluorophores and imaged at 20× using an Akoya Phenocycler platform per manufacturer's instructions. Exposure times for each antibody were consistent across slides (Iba1: 300 ms, HLA-DR: 150 ms, CD68: 200 ms, n-asyn: 200 ms, pTau: 150 ms, MAP2: 150 ms, Amyloid Beta: 150 ms, NeuN: 150 ms). Images were coregistered using Akoya software. Colocalization analysis was performed in QuPath 0.5.1 using manually annotated regions of interest at the CA2 subfield using the same cytoarchitectural boundaries as above brightfield experiments. For each selected annotation, fluorescence intensity values were extracted from the CD68, HLA-DR, and Iba1 channels at full resolution. Only pixels contained within the annotated region were included in the analysis. Channel-specific manual intensity thresholds were applied to define positive signal for each marker, with CD68 thresholded at 2.5 arbitrary units (AU), HLA-DR thresholded at 399.1 AU, and Iba1 thresholded at 45.7 AU for all images. A custom QuPath Groovy script for pixel-based colocalization analysis to generate Manders’ coefficients of colocalization was generated with the aid of ChatGPT model 5.5 to refine code. Manders’ M1 coefficient was defined as the fraction of total threshold-positive CD68 or HLA-DR pixels overlapping with threshold-positive Iba1 pixels and since there can be asymmetry of colocalization, we also calculated a Manders’ M2 as the fraction of total threshold-positive Iba1 pixels overlapping with threshold-positive CD68 or HLA-DR pixels. Furthermore, we also calculated a Manders’ M1 coefficient defined as the fraction of total threshold-positive CD68 pixels with threshold-positive HLA-DR pixels, and the inverse was used to define a Manders’ M2 coefficient [56].

### Statistical analysis

Group comparisons of demographics and clinical data were performed using independent sample two-tailed *t*-tests for continuous measures that follow normality assumptions or Wilcoxon signed-rank test for non-normally distributed continuous measures where indicated. Correlations of %AO were done using non-parametric Spearman rank correlation. To detect outlier data with high %AO in our dataset, we performed a z-score using the scale function in Rstudio (Version 2024.04.2 + 764) for CA2%AO for each marker and defined a statistical outlier as z-score > 3. We then performed sensitivity analyses removing these outlier data. To compare overall patterns of regional burden of immunostain %AO, we used linear mixed effects models, which account for correlations among multiple sample data points within an individual. %AO values were assessed for normality and transformed using natural log. Natural log %AO was used as the dependent variable with fixed effect independent variables of age at death, sex, postmortem interval (PMI) in hours, race (white, non-white, unknown), Consortium to Establish a Registry for Alzheimer’s Disease (CERAD) plaque score (C0, C1, C2, C3), Thal Amyloid Stage (A0, A1, A2, A3), Alzheimer’s tau Braak Stage (B0, B1, B2, B3) [ABC Score], clinical syndrome (PD, PDD, dementia with Lewy bodies), and postmortem interval (PMI) and hippocampal subfield ROI as a fixed factor with the CA2 as the reference strata to identify regional patterns of disease in the linear mixed effects model [36, 47]. A random intercept term was included in linear mixed effects model. Stratified sub-analyses of CA2 CD68 and HLA-DR %AO by tau Braak Stage, Thal Amyloid Stage, and other known co-pathologies were done, by grouping patient measurements of CD68 and HLA-DR %AO in the CA2, by these age-related co-pathologies and comparing score groups using Kruskal–Wallis Test with Dunn’s multiple comparison test. All statistical analyses were performed using Rstudio (Version 2024.04.2 + 764). Data are presented using mean ± standard deviation (SD) unless otherwise indicated with a threshold of *p* = 0.05 for significance based on the hypothesis-driven nature of our regional comparisons.

## Results

### LBD has increased activated microglial phenotypes in the hippocampus relative to PART

**Fig. 1:**
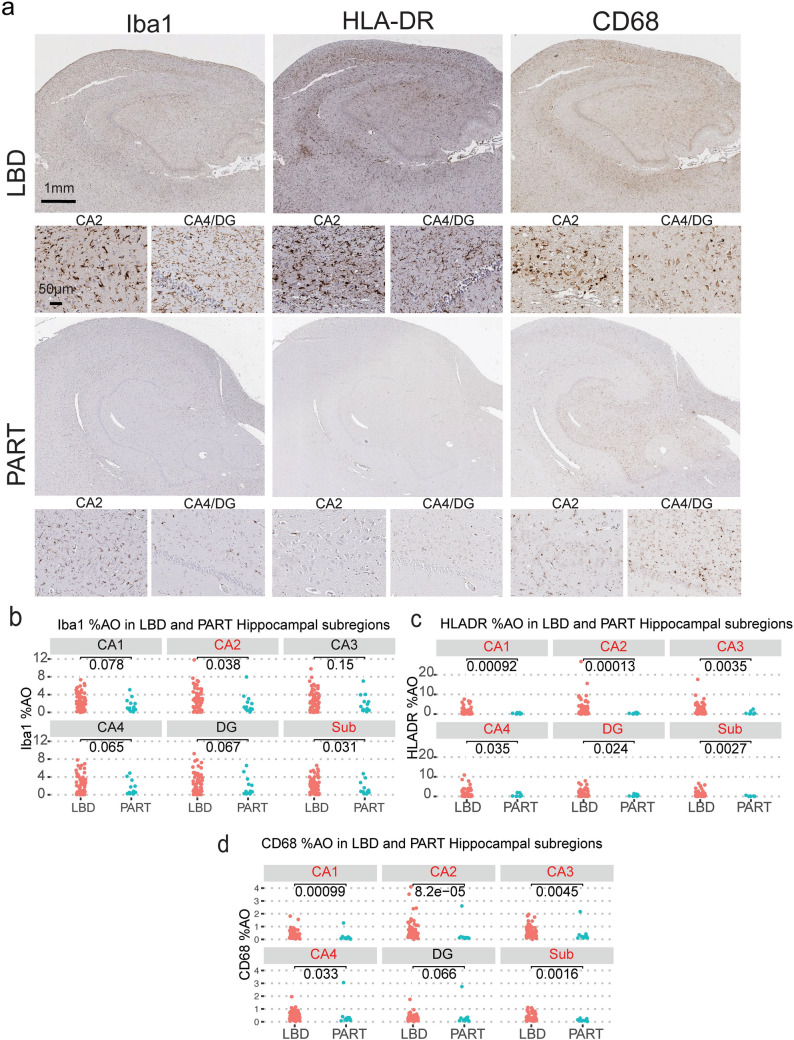
Activated microglial phenotypes in the hippocampus are increased in LBD relative to PART controls. **a** Near-adjacent hippocampal sections stained for Iba1, HLA-DR, and CD68 from an LBD patient and a PART patient. Insets from CA2 and CA4/DG from each image. Scale bars are indicated in image. **b** Comparison of %AO of Iba1, **c** %AO HLA-DR and **d** %AO CD68 between PART and LBD cohorts in each hippocampal subregion. All statistical analyses done using Wilcoxon signed-rank test for nonparametric data. Each data point represents 1 patient sample for that subregion. *n* = 11 for PART cohort in Sub due to damaged tissue precluding measurement. *p*-Values for each comparison are provided on graphs. All statistically significant results demarcated in red font of graph title

We first set out to test if there is increased burden of activated microglial phenotypes in the LBD hippocampal subfields in this relatively “pure” LBD cohort compared to PART normal controls to limit the effect of amyloid on microglial measures [30, 44]. A summary of demographics, neuropathologic findings (including presence of low levels of other age-related pathologies), and clinical data is summarized in Table 1. Briefly, the cohorts were similar in demographics except age, where the PART cohort was younger, with a mean age at death of 64 (Range: 51–83) compared to the LBD cohort which had a mean age at death of 78 (Range: 58–98) (*p* < 0.001). Furthermore, Thal Amyloid Stage (A Score), Alzheimer’s Tau Braak Stage (B Score), and CERAD Plaque Score (C Score) were no different between the PD and PDD groups in the LBD cohort, indicating our matched sampling within these disease subgroups (Supplementary Fig. 3a–c). We found that LBD and PART cohorts had similar staining %AO for the general microglial marker Iba1 in most hippocampal subfields except the CA2 and subiculum where the LBD cohort had slightly increased Iba1%AO (Fig. 1a, b) [41]. In an exploratory analysis, we then evaluated if microglia in the LBD cohort demonstrated greater activated microglial morphological phenotypes between these cohorts by evaluating > 121,000 total microglial cells detected from hippocampal subfields across both cohorts, and we found that microglia in the LBD cohort exhibited increased soma area and critical radius morphologies suggestive of activated phenotypes, but no difference in convex hull area compared to PART (Supplementary Fig. 4a) [71]. We then asked if microglial morphology was altered in specific hippocampal subfields between LBD and PART and found that microglial soma area was higher in the CA2 and Sub subfields of the LBD cohort, but there was no difference in convex hull area or critical radius in any hippocampal subfield (Supplementary Fig. 4b). Next, we set out to evaluate the difference in activated microglial phenotypes between LBD and PART by evaluating for reactivity to the microglial markers of phagocytic activity and antigen presentation, CD68 and HLA-DR %AO, respectively, in the hippocampal subfields. There was increased HLA-DR %AO in LBD compared to PART in all subfields (Fig. 1a, c). CD68%AO was also increased in LBD compared to the PART in all subfields except the DG (Fig. 1a, d). Overall, these data suggest that despite both LBD and PART being characterized by neurodegenerative protein accumulation in the hippocampus, especially in the CA2 subfield, LBD patients exhibit increased measures of activated microglial phenotypes compared to PART in all hippocampal subfields [41, 89].

**Table 1:** Patient demographic and pathological characteristics

	LBD cohort	PART cohort	*p*
Total number of patients included in cohort	62	12	
Median age at autopsy (Range)	78 (58–98)	64 (51–83)	** < 0.001**
Sex [Male] (%)	42 (67.7)	9 (75)	
Median post-mortem interval (range)	11.75 h (2–64)	17 h (6–36)	
Clinical diagnosis	PDD = 39 PD = 19 DLB = 4	Normal = 12*	
Secondary neuropathologic diagnosis	LATE = 4 CVD = 5 PART = 5 AGD = 1 CTE = 1 Tauopathy Unclassifiable = 1	None = 12	
Lewy body disease type	Transitional/Limbic = 40 Diffuse/Neocortical = 22	N/A	
AD Braak stage	B0 = 3 B1 = 52 B2 = 7	B1 = 12	
Amyloid-β plaque score	A0 = 11 A1 = 29 A2 = 14 A3 = 8	A0 = 12	
CERAD score	C0 = 36 C1 = 13 C2 = 5 C3 = 7	C0 = 12	
Race	White = 57 Non-White = 3 Unknown = 2	White = 7 Non-White = 4 Unknown = 1	
DRS total score** median (range)	115 (55–140)	NA	
DRS memory score** median (range)	20 (8–24)	NA	
Neuropsychological testing years prior to death median (range)	1 (0–8)	NA	

*PDD *Parkinson’s disease with Dementia, *PD *Parkinson’s disease, *DLB* Dementia with Lewy Bodies, *AD *Alzheimer’s disease, *LATE *Limbic-predominant age-related TDP-43-associated encephalopathy, *PART* Primary age-related tauopathy, *CTE *Chronic traumatic encephalopathy, *DRS* Dementia Rating Scale. *NA *not available

*One patient had a primary psychiatric diagnosis but no neurodegenerative dementia

**DRS scores were only available for 28 patients

### The CA2 in LBD shows the strongest correlation between n-asyn and activated microglial phenotypes

**Fig. 2:**
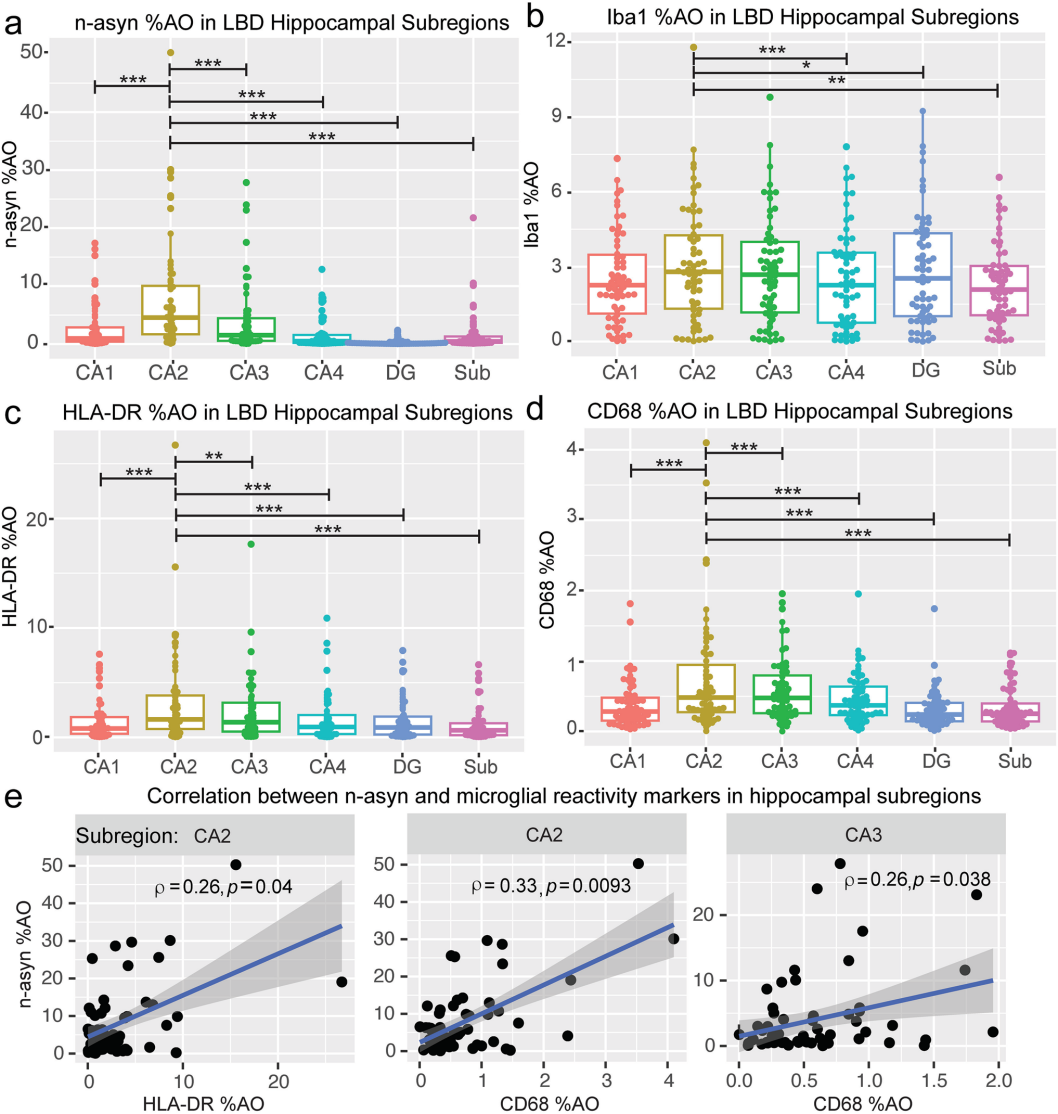
Distribution and correlation of n-asyn and microglial markers in LBD cohort. **a** Box plot of n-asyn %AO, **b** Iba1%AO, **c** HLA-DR %AO, **d** CD68%AO in LBD cohort. Reported *p*-values are derived from natural-log normalized %AO values in linear mixed effects modeling reported in Supplemental Table 1. **e** Spearman rank correlation in indicated subfields of n-asyn %AO with HLA-DR %AO and CD68%AO. * *p* < 0.05, ** *p* < 0.01, *** *p* < 0.001. Blue line is best fit line, and dark gray is confidence interval

To determine the regional distribution and potential association of activated microglial phenotypes in LBD with n-asyn, we tested the regional severity of n-asyn and the microglial markers among the hippocampal subfields in the LBD cohort using linear mixed effects modeling and adjusting for demographics and ADNC-co-pathology (Supplemental Table 1). Similar to previous findings, we found that the CA2 had the greatest n-asyn levels (Fig. 2a; Supplemental Table 1) [21]. For the general microglial marker, Iba1, we found higher %AO in the CA2 compared with the Sub, CA4, and DG, but not the CA1 or 3 (Fig. 2b; Supplemental Table 1). HLA-DR showed the highest %AO in the CA2 relative to all other subregions (Fig. 2c, Supplemental Table 1). CD68 also showed the highest %AO in the CA2 compared with all other hippocampal subregions except the CA3, which had similarly high levels (Fig. 2d, Supplemental Table 1). Importantly, examination of neuropathologic measures, such as Thal Amyloid Stage, Tau Braak Stage, CERAD Score (ABC Score) in our models showed no independent association with any microglial marker (Supplemental Table 1). Among demographic predictors, we did find that male sex associated with increased CD68%AO and that white patients demonstrated higher CD68%AO compared to non-white LBD patients (Supplemental Table 1). We also found that PMI associated with increased Iba1%AO but not HLA-DR and CD68%AO and that age at death associated with decreased Iba1%AO but again not HLA-DR and CD68%AO (Supplemental Table 1).

To determine if the measured burden of activated microglial phenotypes in the LBD hippocampal subfields mirrors n-asyn burden, we tested correlations of n-asyn with each microglial marker within each hippocampal subfield. n-asyn did not correlate with Iba1%AO in any hippocampal subregion, arguing against a linear infiltration of microglia in response to n-asyn burden (Supplementary Fig. 5). Interestingly, both HLA-DR%AO (*ρ* = 0.26, *p* < 0.05) and CD68%AO (*ρ* = 0.33, *p* < 0.01) correlated with n-asyn %AO in the CA2 subfield (Fig. 2e). Within-subfield correlations between n-asyn and HLA-DR did not reach significance in any other subfield (Supplementary Fig. 6a). CD68%AO additionally correlated with n-asyn %AO in the CA3 (*ρ* = 0.26, *p* < 0.05) and the subiculum (*ρ* = 0.3, *p* < 0.05) (Fig. 2e; Supplemental Fig. 7a). Our dataset included a range of biological variation in microglial expression and n-asyn; thus, we hypothesized genetic risk variants known to influence LBD would be associated with higher burdens of pathology in our cohort [15, 20, 81]. We explored genotypes for *APOE* and *GBA* in our cohort and found an *ApoE4* homozygous patient with relatively higher n-asyn %AO (z-score > 4) (*n* = 1, Supplementary Fig. 6a, b) while a patient with no known genetic variant also had high-level CA2 HLA-DR expression (z-score > 5) (*n* = 1, Supplementary Fig. 6a, c). When we excluded these datapoints, we found that there was no longer a statistical correlation between HLA-DR%AO and n-asyn %AO in the CA2 subfield (Supplementary Fig. 6d). Our dataset on CD68 expression in the CA2 had 2 patients with high %AO with a z-score > 3, where 1 was the same *ApoE4* homozygous patient as in the n-asyn dataset and the other had a *GBA* L483P variant (*n* = 1, Supplementary Fig. 7a, b). When we excluded these datapoints, there was still a modest correlation between CD68%AO and n-asyn %AO in the CA2 subfield (Supplementary Fig. 7c). We then asked if tau Braak Stage or copathologies could be driving the correlations of n-asyn %AO with CA2 HLA-DR and CD68%AO. We found that in a stratified sub-analysis of these confounders, there was no difference in CA2 HLA-DR or CD68%AO by Tau Braak stage, Amyloid Thal Phase, or other known copathology (LATE, AGD, etc.). This argues against other age-related copathologies driving the correlation between n-asyn and activated microglial phenotypes (Supplemental Fig. 8a–f). In summation, the CA2 subfield consistently showed the highest amount of staining for IBA1, CD68, and HLA-DR compared with other subfields, and in contrast to the other hippocampal subfields, only the CA2 showed a modest-to-moderate correlation between n-asyn with CD68 and HLA-DR.

### Greater hippocampal n-asyn spatial distribution is associated with worse clinical outcomes

**Fig. 3:**
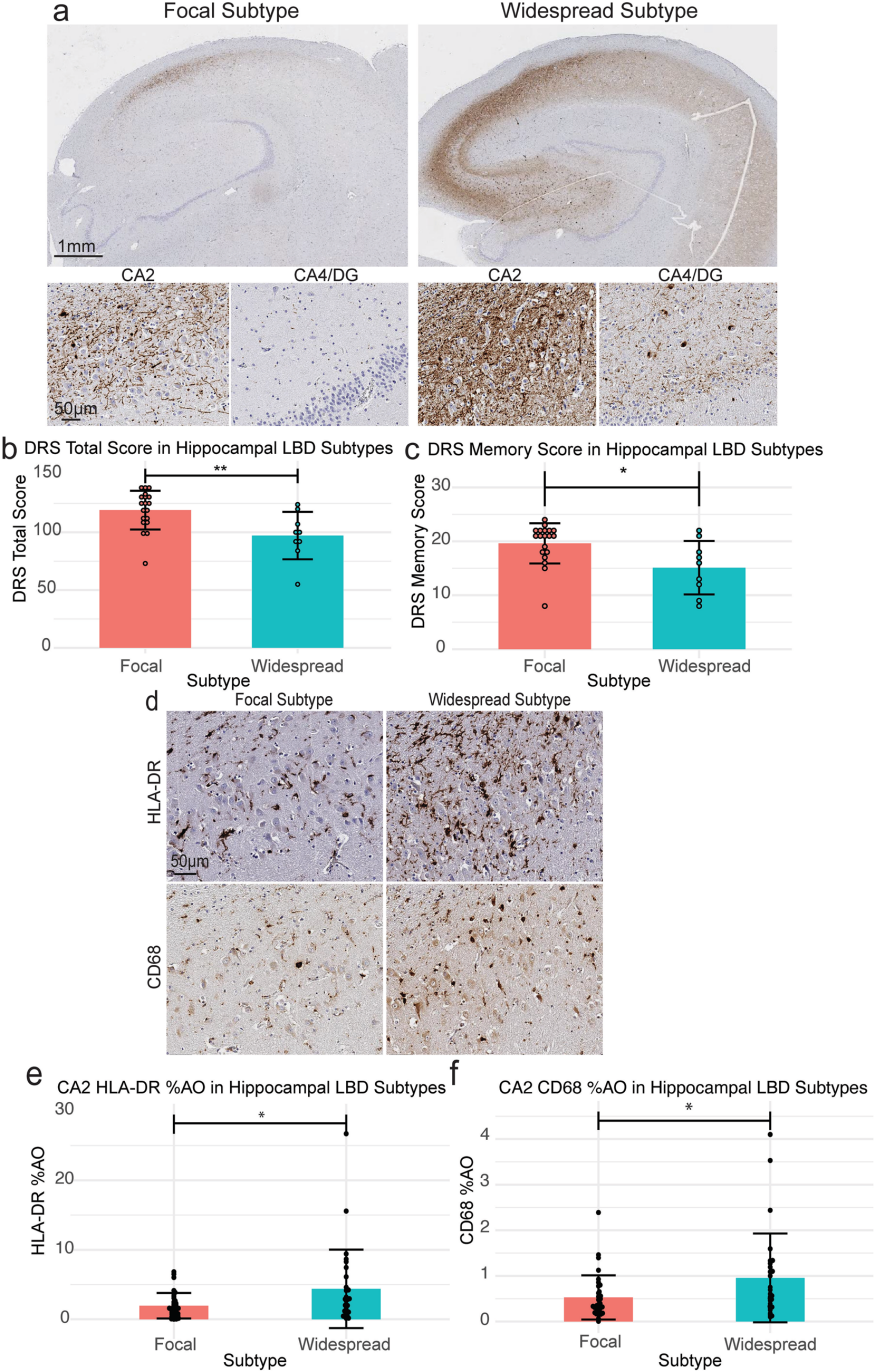
Greater n-asyn distribution in the hippocampus is associated with worse clinical outcomes and greater burden of activated microglial phenotypes in the CA2. **a** Representative images of hippocampal sections stained for n-asyn in the identified Focal and Widespread subtypes. Insets of the CA2 and CA4/DG of each subtype are below the low power images. Scale bars indicated in images. **b** DRS total score and **c** DRS Memory score of patients whose hippocampal sections were categorized into either Focal or Widespread subtypes. Each data point represents the DRS total or DRS memory score from one patient taken at the time interval closest to death. Focal subtype *n* = 19 Widespread subtype *n* = 9. **d** Representative images of HLA-DR and CD68 staining in the CA2 subregion of each subtype. **e** Bar chart of CA2 HLA-DR %AO, **f** CA2 CD68%AO in Focal vs. Widespread patients. Focal subtype *n* = 34 Widespread subtype *n* = 28. All statistical analyses were done using two-tailed Student’s *T*-test. All data are mean ± SD. *p < 0.05, **p < 0.01

Braak staging postulates that greater spatial distribution of n-asyn corresponds to a later disease stage and worse clinical outcomes [12, 13]. Thus, we set out to determine if greater spatial distribution of n-asyn within the intra-hippocampal circuit was also associated with worse clinical outcomes and an increase in activated microglial phenotypes within the hippocampal subfields. Based on visual inspection, we dichotomized patients into two groups. Those that exhibit n-asyn confined within the CA2–3 regions, classified as Focal Subtype, and patients where additional n-asyn was present outside of the CA2–3 region, classified as Widespread Subtype (Fig. 3a). To ensure that subtyping yielded dichotomized groups of n-asyn burden, we compared n-asyn burden in each subfield between these 2 groups and found that the Widespread Subtype exhibited increased n-asyn in all subregions providing neuropathological confirmation of our groupings (Supplemental Fig. 9a–f). This grouping was based on a well-established phosphorylated-α-synuclein S129 epitope as a marker of n-asyn. As further validation of our grouping, we tested semi-quantitative ratings of n-asyn in the hippocampus CA region obtained prospectively at autopsy for neuropathologic diagnosis based on the α-synuclein antibody specific for nitrated post-translational modification, SYN303 [24, 33, 48]. Indeed, we found that n-asyn CA2%AO determined by the MJF-R13 antibody correlated well with SYN303 semi-quantitative ratings (Supplemental Fig. 10a, b). To evaluate for the effect of neuron loss that could confound our LBD widespread vs. focal grouping, we conducted manual neuronal counts in the CA2 between the PART control cohort and Focal and Widespread Subtypes and found no difference in neuronal counts of the CA2 (Supplemental Fig. 11a, b).

Because it is well-established that clinical progression is proportional to n-asyn distribution patterns observed postmortem, we hypothesized that pathology distribution outside of the CA2–3 would correspond to worse clinical outcomes and provide clinical validation of our patient groupings [2, 12, 13]. Within the subset of LBD patients with antemortem DRS scores (*n* = 19 Focal Subtype; *n* = 9 Widespread Subtype), we found that Widespread Subtype patients had a lower DRS total score (Widespread Subtype: 97.11 ± 20.50; Focal Subtype: 119.16 ± 16.78; *p* = 0.006) and DRS memory score (Widespread Subtype: 15.11 ± 4.96; Focal Subtype: 19.63 ± 3.73; *p* = 0.012) compared with the Focal Subtype group (Fig. 3b, c). We also found a non-significant trend for lower MoCA scores (*n* = 17 Focal Subtype; *n* = 8 Widespread Subtype) in Widespread Subtype compared to Focal Subtype patients (Widespread Subtype: 14.75 ± 5.06; Focal Subtype: 18.59 ± 4.01; *p* = 0.052; Supplemental Fig. 12). These data imply that greater spatial distribution of n-asyn within the intra-hippocampal circuit generally correlates with worse clinical progression.

### Activated microglial phenotypes are increased in widespread LBD subtype compared to focal subtype

We next asked if the activated microglial phenotypes are increased in Widespread Subtype patients compared to Focal Subtype patients, and we found that Widespread Subtype patients exhibited increased HLA-DR and CD68%AO compared to Focal Subtype patients, solely in the CA2 subfield (Fig. 3d–f, Supplemental Fig. 13a–j). We next asked if Widespread Subtype patients exhibited altered microglial morphology compared to the Focal Subtype. We found that there was no difference in any microglial morphologic measurement between Focal and Widespread subtypes averaged over the whole hippocampus or in any specific hippocampal subfield (Supplemental Fig. 14a, b). These data illustrate that greater n-asyn levels outside of the vulnerable CA2–3 region are associated with greater burden of activated microglial phenotypes confined to the CA2 despite the presence of n-asyn in hippocampal subfields outside of the CA2 that defined the Widespread Subtype patients.

**Fig. 4:**
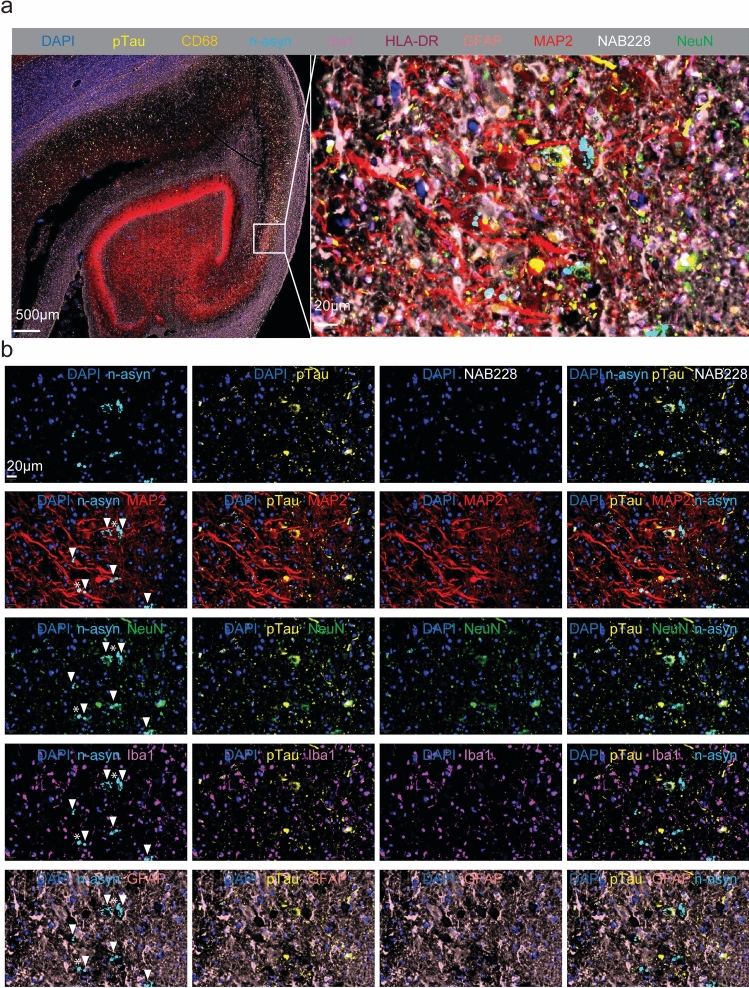
Multiplex immunofluorescence of pathology with cellular markers. **a** Representative image of multiplex immunofluorescence of DAPI, pTau, CD68, n-asyn, Amyloid Beta (NAB228), NeuN, Iba1, HLA-DR, GFAP, and MAP2 of widespread subtype LBD patient hippocampus with inset of CA2 subregion. **b** Selected channels of multiplex immunofluorescence on inset area from (**a**). Colors indicate the specified target in the header of each panel. White arrows point to n-asyn pathological aggregates that colocalize with NeuN and/or MAP2. White asterisks are next to select n-asyn pathological aggregates that do not colocalize with either NeuN or MAP2. Scale bars indicated in images

**Fig. 5:**
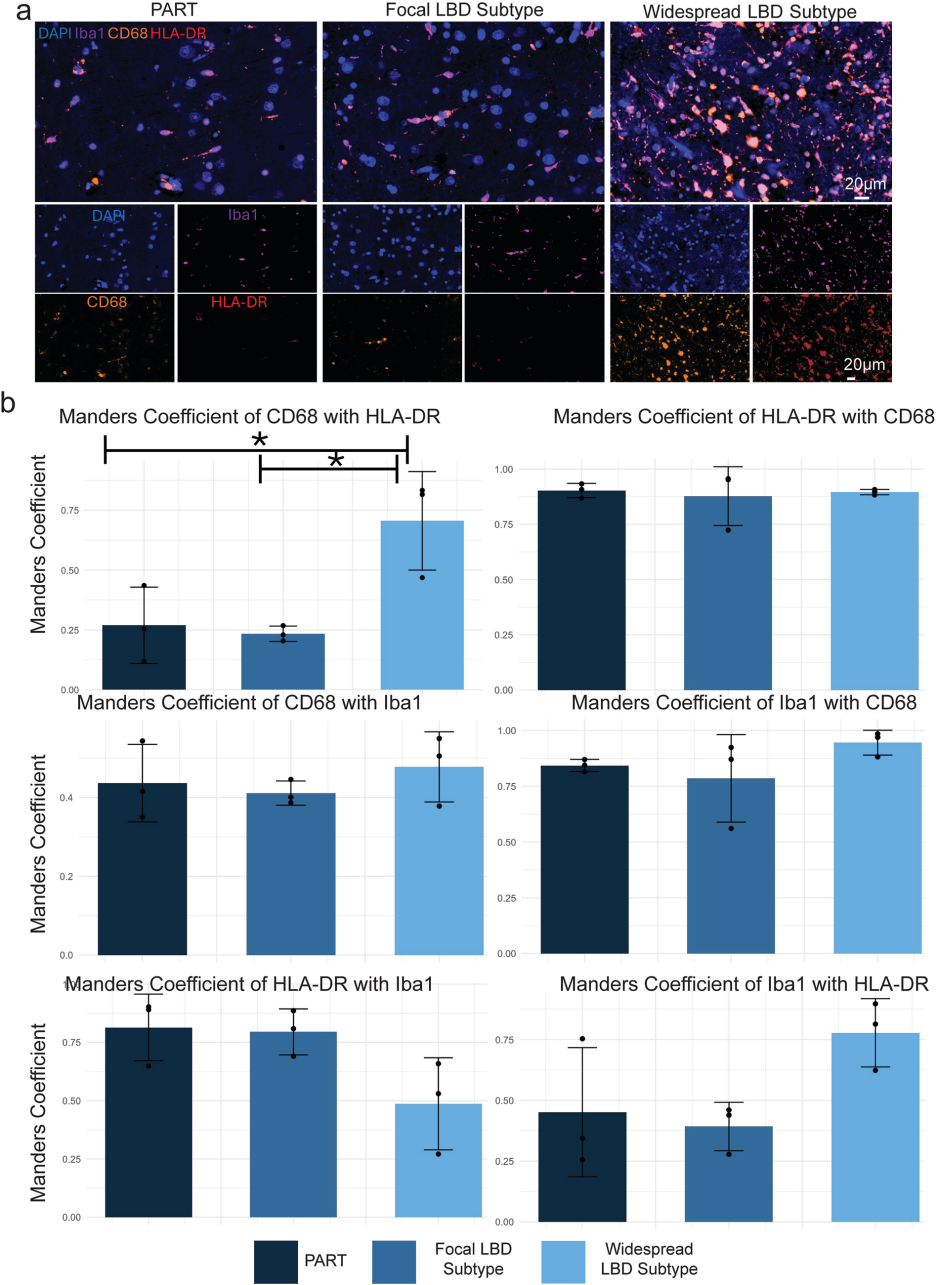
Colocalization of CD68, HLA-DR, and Iba1 in patient subgroups. **a** Representative images of multiplex immunofluorescence of Iba1, CD68, HLA-DR, and DAPI from hippocampal CA2 subregion. Scale bars indicated in image. **b** Bar chart of Manders’ coefficients (M1 and M2) of indicated fluorescent markers in patient groups. Each point is an individual patient. Patient groups labeled by color depicted in legend below graphs. All data are mean ± SD. Statistical analysis done by one-way ANOVA with Tukey’s post hoc analysis. *p < 0.05

Next, using multiplexed immunofluorescent experiments in a subset of patients, we confirmed that the majority of n-asyn was present in neurons by colocalization with MAP2 as a marker of the somatodendritic compartment of neurons, and NeuN, a marker of neuronal cell bodies, but not with Iba1 or GFAP, a marker of astrocytes (Fig. 4a, b). Furthermore, we found that a subset of pyramidal neurons had co-existent n-asyn and pTau inclusions in the CA2 in the LBD cohort, consistent with past work showing co-pathologies are commonly present in the LBD hippocampus (Fig. 4a, b) [18, 25, 28]. Although most n-asyn appeared to colocalize with NeuN or MAP2 suggesting that the aggregates were intraneuronal, a small portion of n-asyn did not colocalize with either MAP2, NeuN, Iba1, or GFAP (Fig. 4b). We then set out to determine if CD68 and HLA-DR upregulation in the CA2 of Widespread Subtype patients occurred within the same microglia or in separate microglial populations. We did this by quantifying the colocalization of CD68, HLA-DR, and Iba1 thresholded pixels in PART, Focal LBD Subtype, and Widespread LBD Subtype patients (*n* = 3 each). We calculated Manders’ coefficient, which is the proportion of pixels from one channel colocalized with a second channel on a 0 (i.e., no overlap) to 1 (i.e., total overlap) scale for each marker-pair accounting for asymmetry in overlap with M1 and M2 coefficients for the reverse marker colocalization [56]. We found that the Widespread LBD Subtype patients demonstrated increased Manders’ colocalization coefficient of CD68 with HLA-DR in the CA2 compared to both Focal LBD subtype and PART patients (*ηp*^2^ = 0.75; 95% CI 0.22–1.00); large effect size; Fig. 5a, b). We did not observe any difference with the reverse colocalization of HLA-DR with CD68 between these groups (p > 0.05). Furthermore, we did not observe any difference in the Manders’ colocalization coefficients of other marker combinations (Fig. 5a, b).

### Activated microglial phenotypes in the CA2 are associated with n-asyn in retrograde parts of the hippocampal circuit

**Fig. 6:**
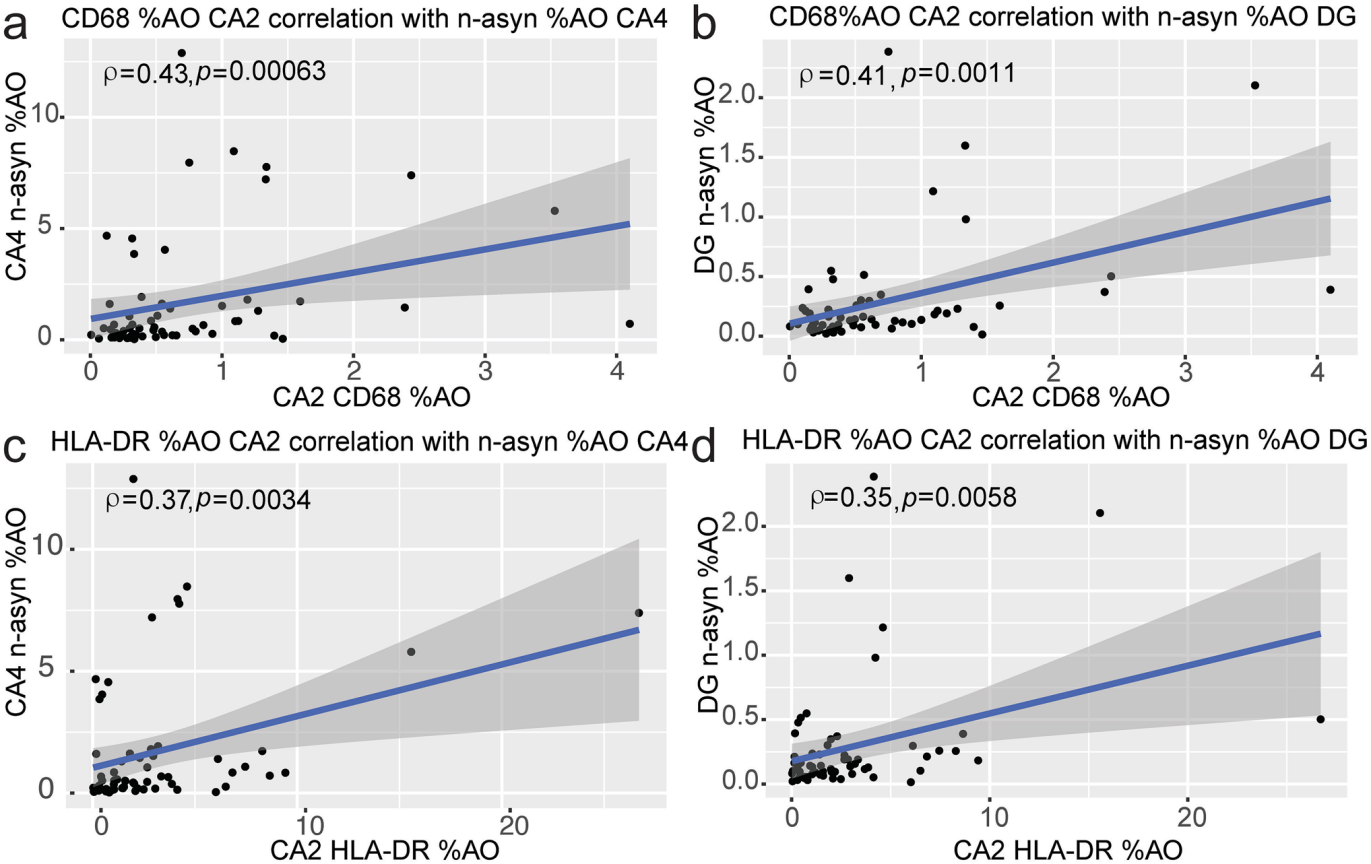
Between-region correlations of activated microglial phenotypes in the CA2 and n-asyn %AO in the CA4 and DG. **a** Spearman rank correlation of CA2 CD68%AO with n-asyn %AO in CA4 and **b** DG. **c** Spearman rank correlation of CA2 HLA-DR %AO with n-asyn %AO in CA4 and **d** DG. Spearman rank correlation coefficient and *p*-values provided on charts. Blue line is best fit line, and dark gray is confidence interval

**Fig. 7:**
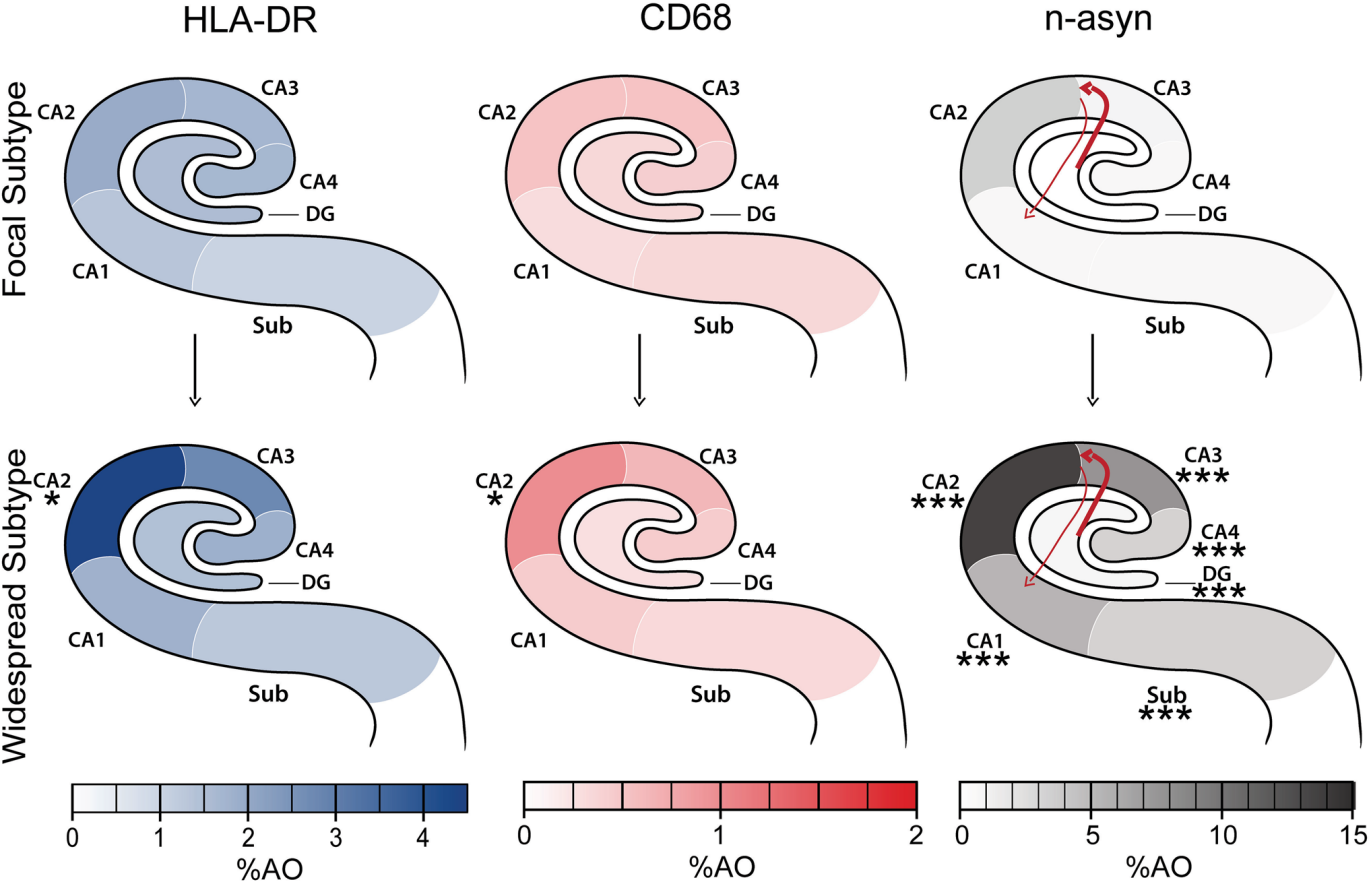
Overview schematic of modeled activated microglial phenotypes and progressive n-asyn accumulation in hippocampal subregions of Focal vs. Widespread subtype LBD patients. Each hippocampal schematic depicts the mean group %AO measurement for each stain (HLA-DR left, CD68 middle, n-asyn right) in each hippocampal subfield in the Focal subtype (top, *n* = 34) and Widespread subtype (bottom, *n* = 28) indicated by color intensity from the associated heat scale intensity bar below each schematic (HLA-DR = blue, CD68 = red, n-asyn = gray). Asterisks denote group-level difference in %AO within subregions between Widespread and Focal subtypes using a two-tailed *t*-test which found increased microglial reactivity to n-asyn exclusively in CA2 subfields despite greater n-asyn spread in all subfields which defined the widespread group compared to the Focal group. *p < 0.05, ***p < 0.001. The pattern of n-asyn spread from CA2 hypothesized via anterograde and retrograde connectivity of the intrahippocampal circuit is depicted within n-asyn schematic (right), where positive correlations of CA2 CD68 and HLA-DR %AO with distal subfields with retrograde connectivity (DG, CA4) are indicated by the thicker line and lack of correlation of these markers in CA2 with subfields with anterograde connectivity (CA1, Sub) are denoted with thin line, suggesting potentially more efficient n-asyn spread via retrograde pathways associated with focal CA2 microglial reactivity

Despite the lack of consistent association between our measures of activated microglial phenotypes and n-asyn burden within each subfield outside of the CA2, we asked if the activated microglial phenotypes in the CA2 correlated with n-asyn burden between hippocampal subfields that are synaptically connected via the intrahippocampal circuit. We found that both CD68%AO and HLA-DR%AO in the CA2 correlated with n-asyn %AO in the CA4 and DG subfields (CD68 CA2 x n-asyn CA4: *ρ* = 0.43, *p* < 0.001; CD68 CA2 x n-asyn DG: *ρ* = 0.41, *p* < 0.01; HLA-DR CA2 x n-asyn CA4: *ρ* = 0.37, *p* < 0.01; HLA-DR CA2 x n-asyn DG: *ρ* = 0.35, *p* < 0.01; Fig. 6a–d). n-asyn levels in neither the subiculum nor CA1 showed a correlation with the measured microglial markers in the CA2 (Supplemental Fig. 15a–d). In the context of hippocampal connectivity, these data show that activated microglial phenotypes in the CA2 are associated with n-asyn levels in the CA4 and DG, which have a retrograde connectivity to the CA2–3 region [39, 53]. n-asyn %AO in the CA1 and Sub, which have anterograde connections with the CA2–3 region, did not correlate with activated microglial phenotypes in the CA2 [53]. Taken together, these data suggest that activated microglial phenotypes in the LBD CA2 hippocampal subfield are associated with hypothesized retrograde temporal progression of n-asyn through the hippocampus (Fig. 7).

## Discussion

We performed a large-scale detailed digital histopathological study using almost 300 slides from >70 autopsied patients to test the hypothesis that activated microglial phenotypes are associated with increasing distribution and severity of n-asyn across the interconnected hippocampal subfields in LBD [89]. Importantly, we found evidence contrary to our hypothesis of an overall correlation of activated microglial phenotypes with n-asyn burden in the hippocampal subfields, where we observed a relatively localized increase in microglial expression of CD68 and HLA-DR in the CA2 subfield which correlated with CA2 n-asyn. This localized increase in activated microglial phenotypes was disease-specific for LBD compared to PART. Moreover, LBD patients with more advanced distribution of hippocampal n-asyn (i.e., Widespread Subtype) have worse cognitive impairment and higher burden of activated microglial phenotypes to the CA2 compared to LBD patients with limited hippocampal n-asyn restricted to CA2–3 region (i.e., Focal Subtype). The activated microglial phenotypes in the CA2 correlated with n-asyn burden in distal subfields with retrograde connectivity but not in subfields with anterograde connectivity. These novel findings have important implications for how microglia contribute to hypothesized progressive spread of n-asyn in the human brain and support a potential role of modulating microglial function as a treatment strategy for LBD.

Previous studies show either no difference or a modest increase in hippocampal microglial activation to n-asyn compared to controls, and lower levels compared to AD [7, 37, 38, 55]. The reasons for these differences are likely multifactorial including various levels of mixed pathology of aging that are common in LBD and normal control cohorts and relatively limited evaluation of individual hippocampal subfields in some studies [30, 37, 38]. Here, we rigorously tested for disease-specificity using a unique study design with strict neuropathological and clinical definitions to define our LBD and normal control PART cohorts to minimize other age-associated co-pathologies (Table 1). Furthermore, we validated the specificity of our results by stratifying our LBD cohort by Braak Tau Stage, Amyloid Thal Phase, and other co-pathologies and found that the measured markers of activated microglial phenotypes, CD68 and HLA-DR, were not affected by these confounders in this relatively “pure” LBD cohort. Finally, we also used multivariate modeling to account for the low-level of AD type pathology and age in our LBD cohort, finding independent associations of n-asyn with the measured microglial markers (Supplemental Table 1). Indeed, pathological findings in aged control cohorts are variable in terms of mixed pathology, and here, we chose to focus our control cohort to only those with PART, which is characterized by pathological tau accumulation that is relatively specific for the hippocampal CA2 subfield with minimal amyloid accumulation [19, 70, 85]. LBD similarly preferentially develops n-asyn in the hippocampal CA2 subfield [21]. Thus, our unique study design allowed us to evaluate for a disease-specific association of n-asyn in LBD on measured activated microglial phenotypes [21]. Interestingly, we found a modest increase in Iba1%AO, the general microglial marker, between the LBD and PART cohorts in the CA2 and Subiculum hippocampal subfields, and we also found that microglia in LBD have increased soma size and critical radius in LBD compared to PART. This modest increase in Iba1%AO in conjunction with the change in morphology argues against a significant infiltration of microglia into the hippocampus due to n-asyn in LBD, although we cannot rule out this possibility completely. Furthermore, we did not evaluate for infiltration of peripheral macrophages into the hippocampus, which can also express Iba1, CD68, and HLA-DR, using a peripheral macrophage marker like CLEC12a, but future work can examine if there is infiltration of peripheral cells into the hippocampus in LBD [29]. Although we controlled for AD Braak tau staging in our multivariate models and in sub-analyses and included markers to two different aspects of microglial activation to obtain converging evidence that indicated the activated microglial phenotypes in the LBD hippocampus are specific to n-asyn, we cannot rule out a contribution of a synergistic effect of tau pathology to the observed hippocampal activated microglial phenotypes (Fig. 1) [11, 49, 84, 89]. Indeed, we previously found focal tau co-pathology in the CA2 associated with n-asyn in relatively pure LBD, and here, we found that n-asyn and tau pathology could co-occur within the same neuronal cell bodies suggesting a potential synergistic effect of n-asyn and tau co-pathology to the development of activated microglial phenotypes [18]. Although our data suggests that most n-asyn pathology was intraneuronal, we did observe a small amount of n-asyn that did not colocalize with neuronal, microglial, or astrocytic markers, in the hippocampal CA2 (Fig. 4). Future work can more comprehensively model tau and n-asyn interactions with neuroinflammation in a larger cohort across LBD, AD, and PART and further refine the subcellular localization of n-asyn.

Interestingly, our data suggest that there may be a range of activated microglial phenotypes within the hippocampus of LBD patients. First, we found measurable microglial morphological differences between LBD and PART only in the CA2 and sub subfields despite an overall more widespread increase in CD68 and HLA-DR expression across most hippocampal subfields. Furthermore, we did not find any differences in microglial morphology in any hippocampal subfield between the Focal and Widespread LBD groups, but there was a measurable increase in CD68 and HLA-DR expression specifically in the CA2 subfield in the Widespread LBD subtype over the Focal subtype. Overall, these data suggest a variety of activated microglial phenotypes are present within the hippocampus in LBD that are not entirely defined by morphology or the canonical activity markers studied here. This suggests that future work utilizing a combination of microglial morphology, microglial proteomic and transcriptomic changes, and neuroanatomical localization can help refine our knowledge regarding the spectrum of activated microglial phenotypes in LBD.

Given the relatively modest increase in Iba1%AO between PART and LBD in this study, our results align with one previous study which found similar levels of hippocampal Iba1 between PD and controls but increased levels of CD68 expression in the CA2 [23]. In that study, there was higher relative CD68 staining in the CA2 compared to other brain regions in healthy controls, and there were also differences in microglial profiles between the hippocampus and substantia nigra pars compacta in PD, suggesting possible regional differences to activated microglial phenotypes [23]. In fact, it is increasingly recognized that microglia adopt differing states, defined as changes in transcriptomic and proteomic signatures, based on their local environment [68]. Recent work in Multiple System Atrophy (MSA), a disorder characterized by the pathologic aggregation of α-synuclein within oligodendrocytes, is associated with a unique microglial subset, and our findings here raise the fascinating possibility of focal dynamic microglial states within select brain areas in LBD [58]. Future work can examine more detailed microglial phenotyping via proteomic and transcriptomic profiles in the hippocampal CA2 subfield across LBD, AD and various aged control patients to further elucidate the microglial state specific to n-asyn.

We also conducted correlational analyses to test the associations of n-asyn with the microglial markers within each subfield and found that the CA2 subfield consistently showed a moderate correlation between n-asyn and microglial reactivity but not with total Iba1%AO (Fig. 2). These data reinforce the specific association of n-asyn in the CA2 subfield with activated microglial phenotypes and not with a global increase in total microglia. Despite the known preferential vulnerability of the hippocampal CA2 subfield to n-asyn, staging systems for LBD have limited description of hypothesized n-asyn progression within the anatomy of hippocampal subfield connectivity [12, 13, 21, 22]. Using quantitative digital data, we developed a limited LBD staging scheme within the hippocampal subfields to test the associations of specific microglial abnormalities with the spread of n-asyn by dichotomizing our LBD group into those with focal α-syn pathology restricted to CA2–3 subregion (Focal Subtype) vs. those with widespread α-syn distribution (Widespread Subtype) (Fig. 3). We validated this staging scheme by confirming the difference in n-asyn %AO between groups was not related to neuron loss (Supplementary Fig. 11) and by testing antemortem cognitive impairment between groups and confirmed that Widespread Subtype patients had worse cognitive impairment closest to death, supporting our model of advancing disease using these groupings. Interestingly, we found higher burden of activated microglial phenotypes associated with n-asyn selectively within the vulnerable CA2 and not within the additional subfields with n-asyn burden that defined the Widespread subtype (Fig. 7). These data suggest that merely the presence of n-asyn within similar neuronal populations, such as the CA1 subfield of the hippocampus, does not elicit the same microglial response as in the CA2. CA2 subfield vulnerability has been implicated in multiple neurodegenerative diseases beyond LBD such as AD, but the mechanism of this vulnerability is unclear [9]. Traditionally, it is difficult to differentiate the CA2 from the CA3 region by morphology alone although the CA2 has unique structural features and is important in social recognition memory [10, 35, 63]. Our data suggest that there is increased burden of activated microglial phenotypes within the CA2 subfield that colocalize CD68 and HLA-DR (Fig. 5) as a possible mechanism of CA2 vulnerability, and past work in primary hippocampal cultures suggests that pyramidal excitatory neurons of the CA2–3 region have higher protein expression levels of α-synuclein compared to other neuronal subpopulations of the hippocampus [54]. Although this work does not fully elucidate the mechanism of the CA2 subfield vulnerability, these data suggest a multifactorial mechanism that includes a greater burden of activated microglial phenotypes.

When we specifically evaluated for associations between n-asyn and activated microglial phenotypes between interconnected subfields, we found that activated microglial phenotypes in the CA2 had moderate correlations with n-asyn in retrograde portions of the intrahippocampal circuit (i.e., CA4 and DG) that exhibit n-asyn in our Widespread Subtype samples and correspond to worse cognitive scores (Fig. 6) [53]. In contrast, n-asyn in anterograde portions of the hippocampal circuit (i.e., CA1 and Sub) did not demonstrate a correlation with the measured CA2 activated microglial phenotypes, although these regions also exhibit more severe n-asyn in our Widespread Subtype classification. Our human autopsy data are cross-sectional in nature, but insights from model systems can inform potential interpretation of n-asyn spread within the human hippocampus. Indeed, both in vitro neuronal cultures and in vivo murine models suggest that n-asyn-like pathology spreads more effectively through retrograde axonal transport, while anterograde transport is slow and inefficient [14, 62, 73, 83]. Furthermore, the injection of misfolded α-synuclein, commonly termed pre-formed fibrils, into the murine hippocampal CA4 preferentially causes n-asyn-like pathology in the CA2/3 region followed by the DG similar to what we observed here in LBD with our staging scheme [54]. Thus, n-asyn in the CA1 and Sub could potentially spread via the less efficient anterograde mechanism or via alternative networks that are less influenced by activated microglial phenotypes in the CA2. Integrated quantitative studies of both human autopsy and model systems can provide more comprehensive staging of n-asyn progression and microglial dynamic states to inform biological staging systems of LBD.

We performed a detailed digital histopathologic study with rigorously validated methods using multiple markers of activated microglial phenotypes and morphologic assessments with careful multivariate modeling to account for potential confounds of aging. However, it is important to consider the potential limitations of our findings. In our unique cohort design, we focused on relatively “pure” LBD patients with limited copathologies. Due to this pathologic focus on pure LBD, our cohort was enriched with clinical PD/PDD and had few dementia with Lewy body patients. Although we accounted for copathologies and clinical presentation in our models, future work inclusive of mixed pathology more common in dementia with Lewy bodies can more comprehensively compare PD/PDD and dementia with Lewy body syndromes.

We focused on the hippocampus due to the well-established connectivity of the intrahippocampal circuit and well-defined neuroanatomy of the hippocampal subfields, but future studies will include larger limbic, neocortical and subcortical networks to determine if other key areas vulnerable to n-asyn share similar activated microglial phenotypes and define the microglial states within those networks. It is notable that we found an association of male sex and non-White race with an increase in CD68 signal in our LBD cohort (Supplemental Table 1). Male sex has been linked to n-asyn in LBD and clinical features of LBD while ethnoracial contributions to LBD are understudied [8, 51, 66]. Our rare autopsy cohort was from a tertiary research center and had limited ethnoracial diversity. Thus, our results may not be fully generalizable to other populations. Future work in population-based cohorts can replicate our findings and further evaluate associations of sex and ancestry with activated microglial phenotypes in LBD. We also found that age and PMI were linked to Iba1%AO but not CD68 and HLA-DR %AO, which reinforces our interpretation that the measured activated microglial phenotypes have an independent association with n-asyn and not confounded by age or PMI, which can affect microglial state [26, 77].

Here we focused on a robust marker of n-asyn pathology (phosphorylated α-synuclein serine 129) to establish associations between LBD pathology and activated microglial states, but n-asyn is well-established to adopt alternate conformations and polymorphs that can vary even within a single patient, which we did not account for in this study [16, 27, 31]. Importantly, we found that alternate measurements of n-asyn, semiquantitative ratings of the SYN303 antibody in the CA region of the hippocampus correlated with the MJFR13 CA2%AO in this study (Supplemental Fig. 10), but alternate conformations or polymorphs may provide additional insights. Furthermore, genetic variants can have alternate effects on microglial states [46]. Interestingly, we found that biological outliers in our data were genetic variants associated with aggressive forms of LBD, such as ApoE4/E4 genotype or presence of a pathogenic *GBA* variant [20, 42, 81]. ApoE4 homozygosity is associated with increased microglial activation in dementia with Lewy bodies in the context of increased amyloid burden, and in mouse models, the induction of copathologies yields greater CD68 expression and peripheral macrophage infiltration [82, 86]. Lastly, neurodegeneration is difficult to accurately assess in the hippocampus and n-asyn may be lost with neurodegeneration [67, 69]. Although, we did not observe overt loss of α-synuclein from severe neurodegeneration in our subtype classification scheme, future experiments using more extensive digital metrics of neuron loss in human tissue sections can be used to more comprehensively test associations of n-asyn, activated microglial phenotypes, and neurodegeneration as these are developed and validated.

Despite these limitations, we present novel data suggesting that increased burden of activated microglial phenotypes can mark a potential epicenter or a neuroinflammatory node of vulnerable brain regions in LBD and help model α-synuclein progression to synaptically connected regions in the human brain. These data will have implications for biomarker studies for n-asyn and neuroinflammation in living patients, which implicate neuroinflammation in early disease [78]. Finally, these postmortem human data provide proof-of-concept to support a therapeutic approach of modulating microglial function in LBD to slow disease progression.

## Supplementary Information

Below is the link to the electronic supplementary material.

Supplementary file1 (PDF 8929 kb)

## Data Availability

All data that were generated or analyzed for this publication are included as part of this publication. Data can be made available from the corresponding authors by request, except where data/results for human subjects cannot be shared due to protection of private health information.

## References

[1] Adamowicz DH, Roy S, Salmon DP, Galasko DR, Hansen LA, Masliah E et al (2017) Hippocampal α-synuclein in dementia with Lewy bodies contributes to memory impairment and is consistent with spread of pathology. J Neurosci 37:1675–1684. 10.1523/JNEUROSCI.3047-16.2016 28039370 10.1523/JNEUROSCI.3047-16.2016 PMC5320602

[2] Adler CH, Beach TG, Zhang N, Shill HA, Driver-Dunckley E, Caviness JN et al (2019) Unified staging system for Lewy body disorders: clinicopathologic correlations and comparison to Braak staging. J Neuropathol Exp Neurol 78:891–899. 10.1093/jnen/nlz080 31504679 10.1093/jnen/nlz080 PMC6751070

[3] Alafuzoff I, Ince PG, Arzberger T, Al-Sarraj S, Bell J, Bodi I et al (2009) Staging/typing of Lewy body related α-synuclein pathology: a study of the BrainNet Europe Consortium. Acta Neuropathol 117:635–652. 10.1007/s00401-009-0523-2 19330340 10.1007/s00401-009-0523-2

[4] Andersen KB, Krishnamurthy A, Just MK, Van Den Berge N, Skjærbæk C, Horsager J et al (2025) Sympathetic and parasympathetic subtypes of body-first Lewy body disease observed in postmortem tissue from prediagnostic individuals. Nat Neurosci 28:925–936. 10.1038/s41593-025-01910-9 40082617 10.1038/s41593-025-01910-9 PMC12081295

[5] Arezoumandan S, Xie SX, Cousins KAQ, Mechanic-Hamilton DJ, Peterson CS, Huang CY et al (2022) Regional distribution and maturation of tau pathology among phenotypic variants of Alzheimer’s disease. Acta Neuropathol. 10.1007/s00401-022-02472-x 35871112 10.1007/s00401-022-02472-x PMC9936795

[6] Attems J, Toledo JB, Walker L, Gelpi E, Gentleman S, Halliday G et al (2021) Neuropathological consensus criteria for the evaluation of Lewy pathology in post-mortem brains: a multi-centre study. Acta Neuropathol 141:159–172. 10.1007/s00401-020-02255-2 33399945 10.1007/s00401-020-02255-2 PMC7847437

[7] Bachstetter AD, Van Eldik LJ, Schmitt FA, Neltner JH, Ighodaro ET, Webster SJ et al (2015) Disease-related microglia heterogeneity in the hippocampus of Alzheimer’s disease, dementia with Lewy bodies, and hippocampal sclerosis of aging. Acta Neuropathol Commun. 10.1186/s40478-015-0209-z 26001591 10.1186/s40478-015-0209-z PMC4489160

[8] Bayram E, Coughlin DG, Banks SJ, Litvan I (2021) Sex differences for phenotype in pathologically defined dementia with Lewy bodies. J Neurol Neurosurg Psychiatry 92:745–750. 10.1136/jnnp-2020-325668 33563809 10.1136/jnnp-2020-325668 PMC8530264

[9] Benarroch E (2026) What is the potential relevance of hippocampal area CA2 in neurologic disorders? Neurology 106:e218096. 10.1212/WNL.0000000000218096 42048628 10.1212/WNL.0000000000218096

[10] Bienkowski MS (2023) Further refining the boundaries of the hippocampus CA2 with gene expression and connectivity: potential subregions and heterogeneous cell types. Hippocampus 33:150–160. 10.1002/hipo.23508 36786207 10.1002/hipo.23508 PMC9987718

[11] Braak H, Braak E (1991) Neuropathological stageing of Alzheimer-related changes. Acta Neuropathol 82:239–259. 10.1007/BF00308809 1759558 10.1007/BF00308809

[12] Braak H, Del Tredici K, Bratzke H, Hamm-Clement J, Sandmann-Keil D, Rüb U (2002) Staging of the intracerebral inclusion body pathology associated with idiopathic Parkinson’s disease (preclinical and clinical stages). J Neurol 249:1–1. 10.1007/s00415-002-1301-4 12528692 10.1007/s00415-002-1301-4

[13] Braak H, Tredici KD, Rüb U, de Vos RAI, Jansen Steur ENH, Braak E (2003) Staging of brain pathology related to sporadic Parkinson’s disease. Neurobiol Aging 24:197–211. 10.1016/S0197-4580(02)00065-9 12498954 10.1016/s0197-4580(02)00065-9

[14] Brahic M, Bousset L, Bieri G, Melki R, Gitler AD (2016) Axonal transport and secretion of fibrillar forms of α-synuclein, Aβ42 peptide and HTTExon 1. Acta Neuropathol 131:539–548. 10.1007/s00401-016-1538-0 26820848 10.1007/s00401-016-1538-0 PMC4789229

[15] Chang D, Nalls MA, Hallgrímsdóttir IB, Hunkapiller J, Van Der Brug M, Cai F et al (2017) A meta-analysis of genome-wide association studies identifies 17 new Parkinson’s disease risk loci. Nat Genet 49:1511–1516. 10.1038/ng.3955 28892059 10.1038/ng.3955 PMC5812477

[16] Chang K, Kim J, Iba M, Park J-H, Beilina A, Syed ZA et al (2025) Differential microglial responses to structurally distinct alpha-synuclein polymorphs. Mol Brain. 10.1186/s13041-025-01256-0 41351016 10.1186/s13041-025-01256-0 PMC12797977

[17] Chhatbar C, Sankowski R, Schulz M, Shimizu T, Schwabenland M, Staszewski O et al (2026) A transcriptomic microglia taxonomy across mouse and human pathologies. Nat Immunol 27:1066–1080. 10.1038/s41590-026-02472-z 41882205 10.1038/s41590-026-02472-z PMC7618987

[18] Coughlin DG, Ittyerah R, Peterson C, Phillips JS, Miller S, Rascovsky K et al (2020) Hippocampal subfield pathologic burden in Lewy body diseases vs Alzheimer’s disease. Neuropathol Appl Neurobiol 46:707–721. 10.1111/nan.12659 32892355 10.1111/nan.12659 PMC7787184

[19] Crary JF, Trojanowski JQ, Schneider JA, Abisambra JF, Abner EL, Alafuzoff I et al (2014) Primary age-related tauopathy (PART): a common pathology associated with human aging. Acta Neuropathol 128:755–766. 10.1007/s00401-014-1349-0 25348064 10.1007/s00401-014-1349-0 PMC4257842

[20] Dickson DW, Heckman MG, Murray ME, Soto AI, Walton RL, Diehl NN et al (2018) *APOE* ε4 is associated with severity of Lewy body pathology independent of Alzheimer pathology. Neurology. 10.1212/WNL.0000000000006212 30143564 10.1212/WNL.0000000000006212 PMC6161556

[21] Dickson DW, Ruan D, Crystal H, Mark MH, Davies P, Kress Y et al (1991) Hippocampal degeneration differentiates diffuse Lewy body disease (DLBD) from Alzheimer’s disease: light and electron microscopic immunocytochemistry of CA2–3 neurites specific to DLBD. Neurology 41:1402–1402. 10.1212/WNL.41.9.1402 1653914 10.1212/wnl.41.9.1402

[22] Dickson DW, Schmidt ML, Lee VM-Y, Zhao M-L, Yen S-H, Trojanowski JQ (1994) Immunoreactivity profile of hippocampal CA2/3 neurites in diffuse Lewy body disease. Acta Neuropathol 87:269–276. 10.1007/BF00296742 7912027 10.1007/BF00296742

[23] Doorn KJ, Moors T, Drukarch B, van de Berg WD, Lucassen PJ, van Dam A-M (2014) Microglial phenotypes and toll-like receptor 2 in the substantia nigra and hippocampus of incidental Lewy body disease cases and Parkinson’s disease patients. Acta Neuropathol Commun 2:90. 10.1186/s40478-014-0090-1 25099483 10.1186/s40478-014-0090-1 PMC4224021

[24] Duda JE, Giasson BI, Mabon ME, Lee VM-Y, Trojanowski JQ (2002) Novel antibodies to synuclein show abundant striatal pathology in Lewy body diseases. Ann Neurol 52:205–210. 10.1002/ana.10279 12210791 10.1002/ana.10279

[25] Duda JE, Giasson BI, Mabon ME, Miller DC, Golbe LI, Lee VM-Y et al (2002) Concurrence of α-synuclein and tau brain pathology in the Contursi kindred. Acta Neuropathol 104:7–11. 10.1007/s00401-002-0563-3 12070658 10.1007/s00401-002-0563-3

[26] Edler MK, Mhatre-Winters I, Richardson JR (2021) Microglia in aging and Alzheimer’s disease: a comparative species review. Cells 10:1138. 10.3390/cells10051138 34066847 10.3390/cells10051138 PMC8150617

[27] Fenyi A, Duyckaerts C, Bousset L, Braak H, Del Tredici K, Melki R et al (2021) Seeding propensity and characteristics of pathogenic αSyn assemblies in formalin-fixed human tissue from the enteric nervous system, olfactory bulb, and brainstem in cases staged for Parkinson’s disease. Cells 10:139. 10.3390/cells10010139 33445653 10.3390/cells10010139 PMC7828121

[28] Fischer DL, VandeVrede L, Seeley WW, Spina S, Wekselman F, Lago AL et al (2024) Tackling selective neuronal vulnerability: subnuclei involvement of amygdala-predominant Lewy body disease in early-onset Alzheimer’s disease. Alzheimers Dement 20:e093381. 10.1002/alz.093381

[29] Forbes LH, Miron VE (2022) Monocytes in central nervous system remyelination. Glia 70:797–807. 10.1002/glia.24111 34708884 10.1002/glia.24111

[30] Fruhwürth S, Zetterberg H, Paludan SR (2024) Microglia and amyloid plaque formation in Alzheimer’s disease – evidence, possible mechanisms, and future challenges. J Neuroimmunol 390:578342. 10.1016/j.jneuroim.2024.578342 38640827 10.1016/j.jneuroim.2024.578342

[31] Fujiwara H, Hasegawa M, Dohmae N, Kawashima A, Masliah E, Goldberg MS et al (2002) α-Synuclein is phosphorylated in synucleinopathy lesions. Nat Cell Biol 4:160–164. 10.1038/ncb748 11813001 10.1038/ncb748

[32] Geijs D, Intezar M, Litjens GJS, Van Der Laak J (2018) Automatic color unmixing of IHC stained whole slide images. In: Gurcan MN, Tomaszewski JE (eds) Medical imaging 2018: digital pathology. SPIE, Houston, p 20

[33] Giasson BI, Duda JE, Murray IVJ, Chen Q, Souza JM, Hurtig HI et al (2000) Oxidative damage linked to neurodegeneration by selective α-synuclein nitration in synucleinopathy lesions. Science 290:985–989. 10.1126/science.290.5493.985 11062131 10.1126/science.290.5493.985

[34] Goedert M, Spillantini MG, Del Tredici K, Braak H (2013) 100 years of Lewy pathology. Nat Rev Neurol 9:13–24. 10.1038/nrneurol.2012.242 23183883 10.1038/nrneurol.2012.242

[35] Hitti FL, Siegelbaum SA (2014) The hippocampal CA2 region is essential for social memory. Nature 508:88–92. 10.1038/nature13028 24572357 10.1038/nature13028 PMC4000264

[36] Hyman BT, Phelps CH, Beach TG, Bigio EH, Cairns NJ, Carrillo MC et al (2012) National Institute on Aging-Alzheimer’s Association guidelines for the neuropathologic assessment of Alzheimer’s disease. Alzheimers Dement 8:1–13. 10.1016/j.jalz.2011.10.007 22265587 10.1016/j.jalz.2011.10.007 PMC3266529

[37] Imamura K, Hishikawa N, Ono K, Suzuki H, Sawada M, Nagatsu T et al (2005) Cytokine production of activated microglia and decrease in neurotrophic factors of neurons in the hippocampus of Lewy body disease brains. Acta Neuropathol 109:141–150. 10.1007/s00401-004-0919-y 15619128 10.1007/s00401-004-0919-y

[38] Imamura K, Hishikawa N, Sawada M, Nagatsu T, Yoshida M, Hashizume Y (2003) Distribution of major histocompatibility complex class II-positive microglia and cytokine profile of Parkinson’s disease brains. Acta Neuropathol 106:518–526. 10.1007/s00401-003-0766-2 14513261 10.1007/s00401-003-0766-2

[39] Insausti R, Muñoz M (2001) Cortical projections of the non‐entorhinal hippocampal formation in the cynomolgus monkey (*Macaca fascicularis)*. Eur J Neurosci 14:435–451. 10.1046/j.0953-816x.2001.01662.x 11553294 10.1046/j.0953-816x.2001.01662.x

[40] Irwin DJ, Grossman M, Weintraub D, Hurtig HI, Duda JE, Xie SX et al (2017) Neuropathological and genetic correlates of survival and dementia onset in synucleinopathies: a retrospective analysis. Lancet Neurol 16:55–65. 10.1016/S1474-4422(16)30291-5 27979356 10.1016/S1474-4422(16)30291-5 PMC5181646

[41] Ito D, Imai Y, Ohsawa K, Nakajima K, Fukuuchi Y, Kohsaka S (1998) Microglia-specific localisation of a novel calcium binding protein, Iba1. Mol Brain Res 57:1–9. 10.1016/S0169-328X(98)00040-0 9630473 10.1016/s0169-328x(98)00040-0

[42] Kaivola K, Shah Z, Chia R, Black SE, Gan-Or Z, Keith J et al (2022) Genetic evaluation of dementia with Lewy bodies implicates distinct disease subgroups. Brain 145:1757–1762. 10.1093/brain/awab402 35381062 10.1093/brain/awab402 PMC9423712

[43] Kayasandik C, Guo K, Labate D (2019) Directional multiscale representations and applications in digital neuron reconstruction. J Comput Appl Math 349:482–493. 10.1016/j.cam.2018.09.003

[44] Keren-Shaul H, Spinrad A, Weiner A, Matcovitch-Natan O, Dvir-Szternfeld R, Ulland TK et al (2017) A unique microglia type associated with restricting development of Alzheimer’s disease. Cell 169:1276-1290.e17. 10.1016/j.cell.2017.05.018 28602351 10.1016/j.cell.2017.05.018

[45] Kim B, Mikytuck B, Suh E, Gibbons GS, Van Deerlin VM, Vaishnavi SN et al (2021) Tau immunotherapy is associated with glial responses in FTLD-tau. Acta Neuropathol 142:243–257. 10.1007/s00401-021-02318-y 33950293 10.1007/s00401-021-02318-y PMC8270872

[46] Krzisch M, Yuan B, Chen W, Osaki T, Fu D, Garrett-Engele CM et al (2025) The A53T mutation in α-synuclein enhances proinflammatory activation in human microglia upon inflammatory stimulus. Biol Psychiatry 97:730–742. 10.1016/j.biopsych.2024.07.011 39029776 10.1016/j.biopsych.2024.07.011

[47] Laird NM, Ware JH (1982) Random-effects models for longitudinal data. Biometrics 38:963–974 7168798

[48] Lashuel HA, Mahul-Mellier A-L, Novello S, Hegde RN, Jasiqi Y, Altay MF et al (2022) Revisiting the specificity and ability of phospho-S129 antibodies to capture alpha-synuclein biochemical and pathological diversity. NPJ Parkinsons Dis 8:136. 10.1038/s41531-022-00388-7 36266318 10.1038/s41531-022-00388-7 PMC9584898

[49] Lee YB, Nagai A, Kim SU (2002) Cytokines, chemokines, and cytokine receptors in human microglia. J Neurosci Res 69:94–103. 10.1002/jnr.10253 12111820 10.1002/jnr.10253

[50] Leverenz JB, Hamilton R, Tsuang DW, Schantz A, Vavrek D, Larson EB et al (2008) Research article: empiric refinement of the pathologic assessment of Lewy‐related pathology in the dementia patient. Brain Pathol 18:220–224. 10.1111/j.1750-3639.2007.00117.x 18241240 10.1111/j.1750-3639.2007.00117.x PMC2689097

[51] Lewy Body Dementia Association Community Engagement Working Group, Bayram E, Holden SK, Fullard M, Armstrong MJ (2023) Race and ethnicity in Lewy body dementia: a narrative review. J Alzheimers Dis 94:861–878. 10.3233/JAD-230207 37355902 10.3233/JAD-230207 PMC10448838

[52] Leyh J, Paeschke S, Mages B, Michalski D, Nowicki M, Bechmann I et al (2021) Classification of microglial morphological phenotypes using machine learning. Front Cell Neurosci 15:701673. 10.3389/fncel.2021.701673 34267628 10.3389/fncel.2021.701673 PMC8276040

[53] Lisman JE (1999) Relating hippocampal circuitry to function. Neuron 22:233–242. 10.1016/S0896-6273(00)81085-5 10069330 10.1016/s0896-6273(00)81085-5

[54] Luna E, Decker SC, Riddle DM, Caputo A, Zhang B, Cole T et al (2018) Differential α-synuclein expression contributes to selective vulnerability of hippocampal neuron subpopulations to fibril-induced toxicity. Acta Neuropathol 135:855–875. 10.1007/s00401-018-1829-8 29502200 10.1007/s00401-018-1829-8 PMC5955788

[55] Mackenzie IRA (2000) Activated microglia in dementia with Lewy bodies. Neurology 55:132–134. 10.1212/WNL.55.1.132 10891923 10.1212/wnl.55.1.132

[56] Manders EMM, Verbeek FJ, Aten JA (1993) Measurement of co-localization of objects in dual-colour confocal images. J Microsc 169:375–382. 10.1111/j.1365-2818.1993.tb03313.x 33930978 10.1111/j.1365-2818.1993.tb03313.x

[57] Mastenbroek SE, Vogel JW, Collij LE, Serrano GE, Tremblay C, Young AL et al (2024) Disease progression modelling reveals heterogeneity in trajectories of Lewy-type α-synuclein pathology. Nat Commun 15:5133. 10.1038/s41467-024-49402-x 38879548 10.1038/s41467-024-49402-x PMC11180185

[58] Matsuse D, Yamaguchi H, Harada M, Kira Y, Nishimura Y, Masaki K et al (2026) A unique microglia subset associated with aggressive α-synucleinopathy uncovered in a rapidly progressive multiple system atrophy cerebellar type model. Neurobiol Dis 218:107206. 10.1016/j.nbd.2025.107206 41325908 10.1016/j.nbd.2025.107206

[59] McGeer PL, Itagaki S, Boyes BE, McGeer EG (1988) Reactive microglia are positive for HLA-DR in the substantia nigra of Parkinson’s and Alzheimer’s disease brains. Neurology 38:1285–1285. 10.1212/WNL.38.8.1285 3399080 10.1212/wnl.38.8.1285

[60] McGeer PL, McGeer EG (2008) Glial reactions in Parkinson’s disease. Mov Disord 23:474–483. 10.1002/mds.21751 18044695 10.1002/mds.21751

[61] McKeith IG, Galasko D, Kosaka K, Perry EK, Dickson DW, Hansen LA et al (1996) Consensus guidelines for the clinical and pathologic diagnosis of dementia with Lewy bodies (DLB): report of the consortium on DLB international workshop. Neurology 47:1113–1124. 10.1212/WNL.47.5.1113 8909416 10.1212/wnl.47.5.1113

[62] Mezias C, Rey N, Brundin P, Raj A (2020) Neural connectivity predicts spreading of alpha-synuclein pathology in fibril-injected mouse models: involvement of retrograde and anterograde axonal propagation. Neurobiol Dis 134:104623. 10.1016/j.nbd.2019.104623 31628991 10.1016/j.nbd.2019.104623 PMC7138530

[63] Middleton SJ, McHugh TJ (2020) CA2: a highly connected intrahippocampal relay. Annu Rev Neurosci 43:55–72. 10.1146/annurev-neuro-080719-100343 31874067 10.1146/annurev-neuro-080719-100343

[64] Montine TJ, Phelps CH, Beach TG, Bigio EH, Cairns NJ, Dickson DW et al (2012) National Institute on Aging–Alzheimer’s Association guidelines for the neuropathologic assessment of Alzheimer’s disease: a practical approach. Acta Neuropathol 123:1–11. 10.1007/s00401-011-0910-3 22101365 10.1007/s00401-011-0910-3 PMC3268003

[65] Nalls MA, Blauwendraat C, Vallerga CL, Heilbron K, Bandres-Ciga S, Chang D et al (2019) Identification of novel risk loci, causal insights, and heritable risk for Parkinson’s disease: a meta-analysis of genome-wide association studies. Lancet Neurol 18:1091–1102. 10.1016/S1474-4422(19)30320-5 31701892 10.1016/S1474-4422(19)30320-5 PMC8422160

[66] Nelson PT, Schmitt FA, Jicha GA, Kryscio RJ, Abner EL, Smith CD et al (2010) Association between male gender and cortical Lewy body pathology in large autopsy series. J Neurol 257:1875–1881. 10.1007/s00415-010-5630-4 20563821 10.1007/s00415-010-5630-4 PMC3040648

[67] Osterberg VR, Spinelli KJ, Weston LJ, Luk KC, Woltjer RL, Unni VK (2015) Progressive aggregation of Alpha-Synuclein and selective degeneration of Lewy inclusion-bearing neurons in a mouse model of Parkinsonism. Cell Rep 10:1252–1260. 10.1016/j.celrep.2015.01.060 25732816 10.1016/j.celrep.2015.01.060 PMC4351119

[68] Paolicelli RC, Sierra A, Stevens B, Tremblay M-E, Aguzzi A, Ajami B et al (2022) Microglia states and nomenclature: a field at its crossroads. Neuron 110:3458–3483. 10.1016/j.neuron.2022.10.020 36327895 10.1016/j.neuron.2022.10.020 PMC9999291

[69] Patterson JR, Duffy MF, Kemp CJ, Howe JW, Collier TJ, Stoll AC et al (2019) Time course and magnitude of alpha-synuclein inclusion formation and nigrostriatal degeneration in the rat model of synucleinopathy triggered by intrastriatal α-synuclein preformed fibrils. Neurobiol Dis 130:104525. 10.1016/j.nbd.2019.104525 31276792 10.1016/j.nbd.2019.104525 PMC6701176

[70] Power MC, Mormino E, Soldan A, James BD, Yu L, Armstrong NM et al (2018) Combined neuropathological pathways account for age-related risk of dementia. Ann Neurol 84:10–22. 10.1002/ana.25246 29944741 10.1002/ana.25246 PMC6119518

[71] Reddaway J, Richardson PE, Bevan RJ, Stoneman J, Palombo M (2023) Microglial morphometric analysis: so many options, so little consistency. Front Neuroinform 17:1211188. 10.3389/fninf.2023.1211188 37637472 10.3389/fninf.2023.1211188 PMC10448193

[72] Rosin PL (2001) Unimodal thresholding. Pattern Recogn 34:2083–2096. 10.1016/S0031-3203(00)00136-9

[73] Schaser AJ, Stackhouse TL, Weston LJ, Kerstein PC, Osterberg VR, López CS et al (2020) Trans-synaptic and retrograde axonal spread of Lewy pathology following pre-formed fibril injection in an in vivo A53T alpha-synuclein mouse model of synucleinopathy. Acta Neuropathol Commun 8:150. 10.1186/s40478-020-01026-0 32859276 10.1186/s40478-020-01026-0 PMC7456087

[74] Simuni T, Chahine LM, Poston K, Brumm M, Buracchio T, Campbell M et al (2024) A biological definition of neuronal α-synuclein disease: towards an integrated staging system for research. Lancet Neurol 23:178–190. 10.1016/S1474-4422(23)00405-2 38267190 10.1016/S1474-4422(23)00405-2

[75] Smajić S, Prada-Medina CA, Landoulsi Z, Ghelfi J, Delcambre S, Dietrich C et al (2022) Single-cell sequencing of human midbrain reveals glial activation and a Parkinson-specific neuronal state. Brain 145:964–978. 10.1093/brain/awab446 34919646 10.1093/brain/awab446 PMC9050543

[76] Spillantini MG, Schmidt ML, Lee VM-Y, Trojanowski JQ, Jakes R, Goedert M (1997) α-Synuclein in Lewy bodies. Nature 388:839–840. 10.1038/42166 9278044 10.1038/42166

[77] St-Pierre M-K, Šimončičová E, Carrier M, Tremblay M-È (2023) Microglia in human postmortem brain samples: quantitative ultrastructural analysis of scanning electron microscopy images. In: Chun J (ed) Alzheimer’s disease. Springer, New York, pp 63–85 10.1007/978-1-0716-2655-9_4 36399265

[78] Surendranathan A, Su L, Mak E, Passamonti L, Hong YT, Arnold R et al (2018) Early microglial activation and peripheral inflammation in dementia with Lewy bodies. Brain 141:3415–3427. 10.1093/brain/awy265 30403785 10.1093/brain/awy265 PMC6262214

[79] Toledo JB, Van Deerlin VM, Lee EB, Suh E, Baek Y, Robinson JL et al (2014) A platform for discovery: The University of Pennsylvania Integrated Neurodegenerative Disease Biobank. Alzheimer’s Dementia 10:477. 10.1016/j.jalz.2013.06.003 23978324 10.1016/j.jalz.2013.06.003 PMC3933464

[80] Trainor AR, MacDonald DS, Penney J (2024) Microglia: roles and genetic risk in Parkinson’s disease. Front Neurosci 18:1506358. 10.3389/fnins.2024.1506358 39554849 10.3389/fnins.2024.1506358 PMC11564156

[81] Tsuang D, Leverenz JB, Lopez OL, Hamilton RL, Bennett DA, Schneider JA et al (2012) GBA mutations increase risk for Lewy body disease with and without Alzheimer disease pathology. Neurology 79:1944–1950. 10.1212/WNL.0b013e3182735e9a 23035075 10.1212/WNL.0b013e3182735e9a PMC3484986

[82] Van Wetering J, Geut H, Bol JJ, Galis Y, Timmermans E, Twisk JWR et al (2025) Correction: Neuroinflammation is associated with Alzheimer’s disease co-pathology in dementia with Lewy bodies. Acta Neuropathol Commun 13:250. 10.1186/s40478-025-02169-8 41366818 10.1186/s40478-025-02169-8 PMC12690777

[83] Vroman R, De Lichtervelde L, Singh Dolt K, Robertson G, Kriek M, Barbato M et al (2025) A high-fidelity microfluidic platform reveals retrograde propagation as the main mechanism of α-synuclein spread in human neurons. NPJ Parkinsons Dis 11:80. 10.1038/s41531-025-00936-x 40254612 10.1038/s41531-025-00936-x PMC12009960

[84] Walker DG, Lue L-F (2015) Immune phenotypes of microglia in human neurodegenerative disease: challenges to detecting microglial polarization in human brains. Alz Res Therapy 7:56. 10.1186/s13195-015-0139-9 10.1186/s13195-015-0139-9 PMC4543480 26286145

[85] Walker JM, Richardson TE, Farrell K, Iida MA, Foong C, Shang P et al (2021) Early selective vulnerability of the CA2 hippocampal subfield in primary age-related tauopathy. J Neuropathol Exp Neurol 80:102–111. 10.1093/jnen/nlaa153 33367843 10.1093/jnen/nlaa153 PMC8453611

[86] Webster JM, Yang Y-T, Miller AT, Zane A, Scholz K, Stone WJ et al (2024) Tau, amyloid-β and α-synuclein co-pathologies synergistically enhance neuroinflammation and neuropathology. bioRxiv. 10.1101/2024.10.13.61810139677620

[87] Weintraub D, Posavi M, Fontanillas P, Tropea TF, Mamikonyan E, Suh E et al (2022) Genetic prediction of impulse control disorders in Parkinson’s disease. Ann Clin Transl Neurol 9:936–949. 10.1002/acn3.51569 35762106 10.1002/acn3.51569 PMC9268896

[88] Xie SX, Baek Y, Grossman M, Arnold SE, Karlawish J, Siderowf A et al (2011) Building an integrated neurodegenerative disease database at an academic health center. Alzheimer’s Dementia. 10.1016/j.jalz.2010.08.233 21784346 10.1016/j.jalz.2010.08.233 PMC3145967

[89] Zrzavy T, Höftberger R, Berger T, Rauschka H, Butovsky O, Weiner H et al (2019) Pro-inflammatory activation of microglia in the brain of patients with sepsis. Neuropathol Appl Neurobiol 45:278–290. 10.1111/nan.12502 29804289 10.1111/nan.12502 PMC6487964

