# Supplementary material for: Activated microglial phenotypes in the hippocampal CA2 subfield are implicated in Lewy body disease progression
Source: Acta Neuropathol. 2026 Aug 8;152(1):17. doi: 10.1007/s00401-026-03065-8 (PMC13452847; doi:10.1007/s00401-026-03065-8)
Supplement: Supplementary file 1 [file 401_2026_3065_MOESM1_ESM.pdf]

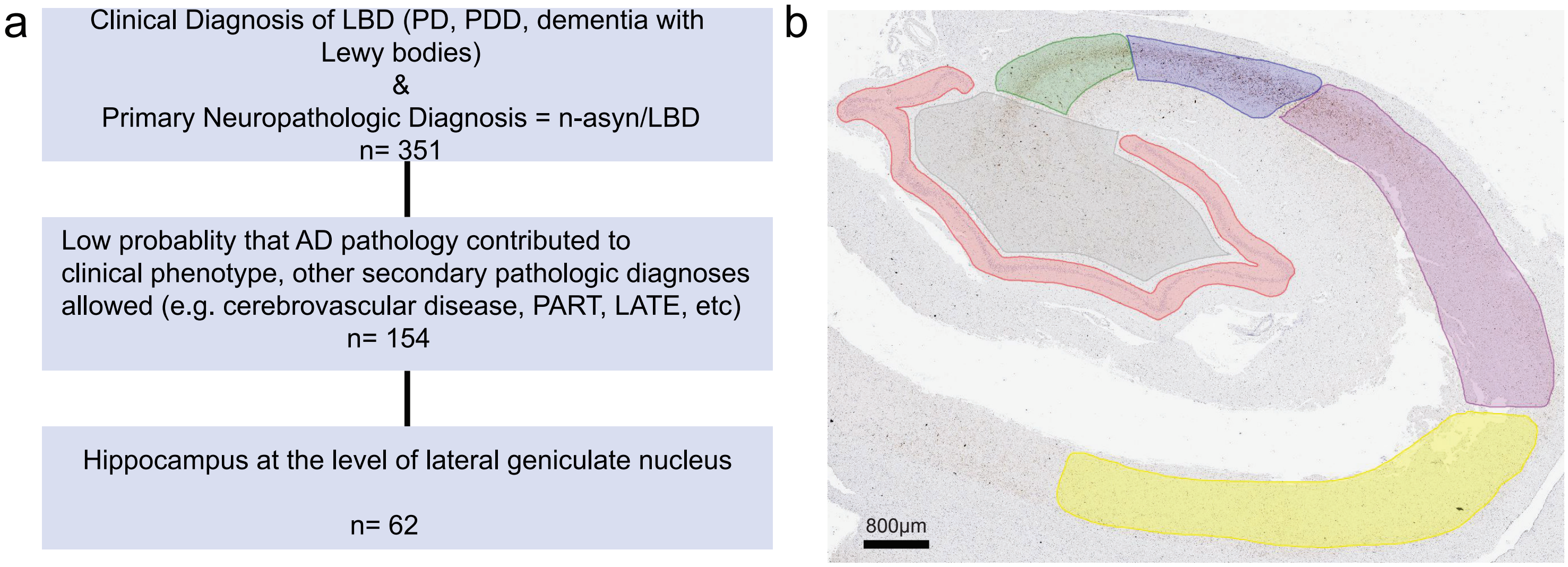

Supplemental Figure 1. Study design and hippocampal annotation. a) The flow diagram describes the inclusion and exclusion criteria for selecting patient samples for the LBD cohort. **b)** Hippocampal subfield annotations of a LBD patient sample stained for n-asyn. Red = DG, Grey = CA4, Green = CA3, Blue = CA2, Maroon = CA1, Yellow = Sub. Scale bar indicated in image.

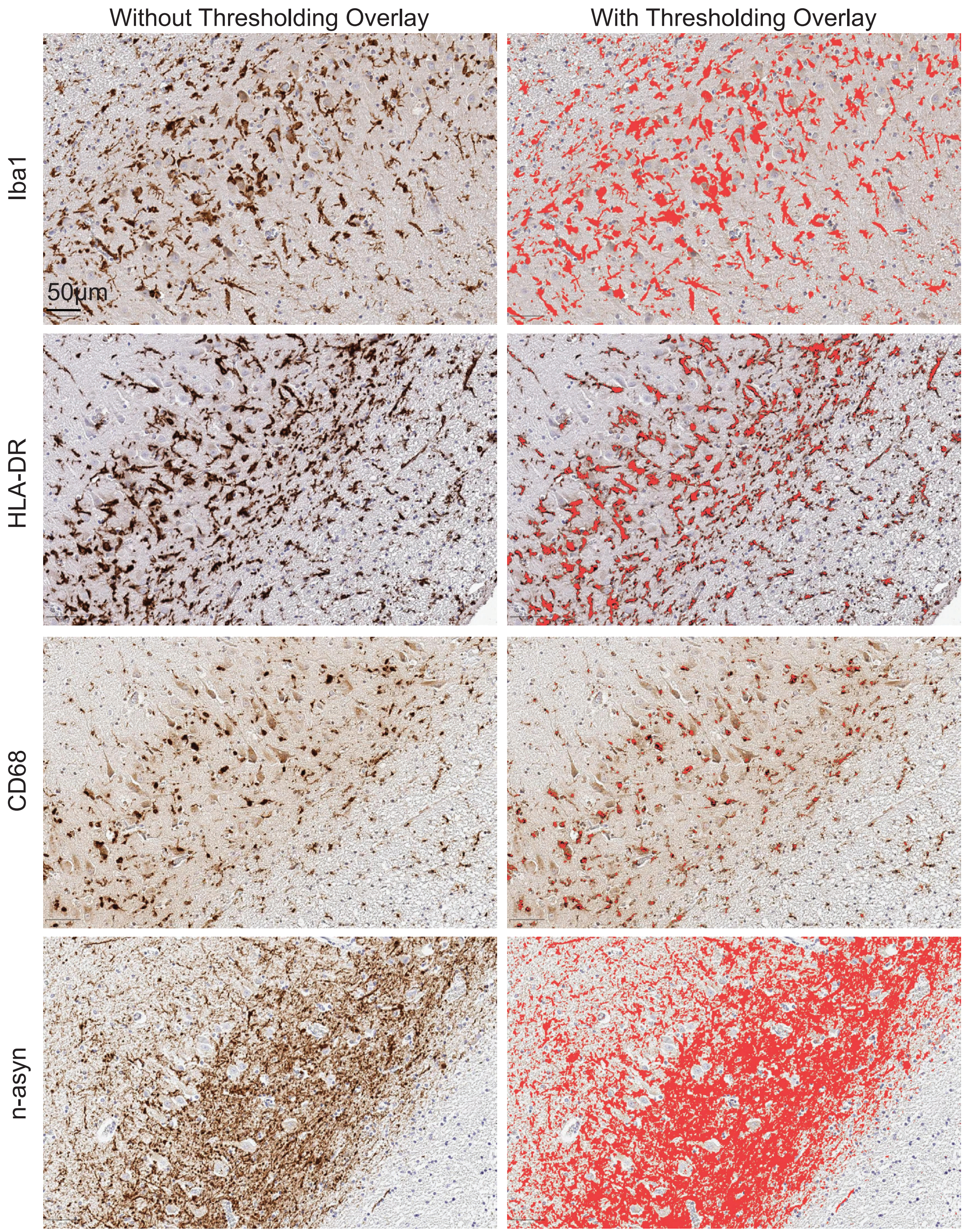

Supplemental Figure 2 Example Thresholding of stained markers. Representative images of the CA2 from the same patient of the indicated stained markers with and without thresholding overlay. Scale bar indicated on image.

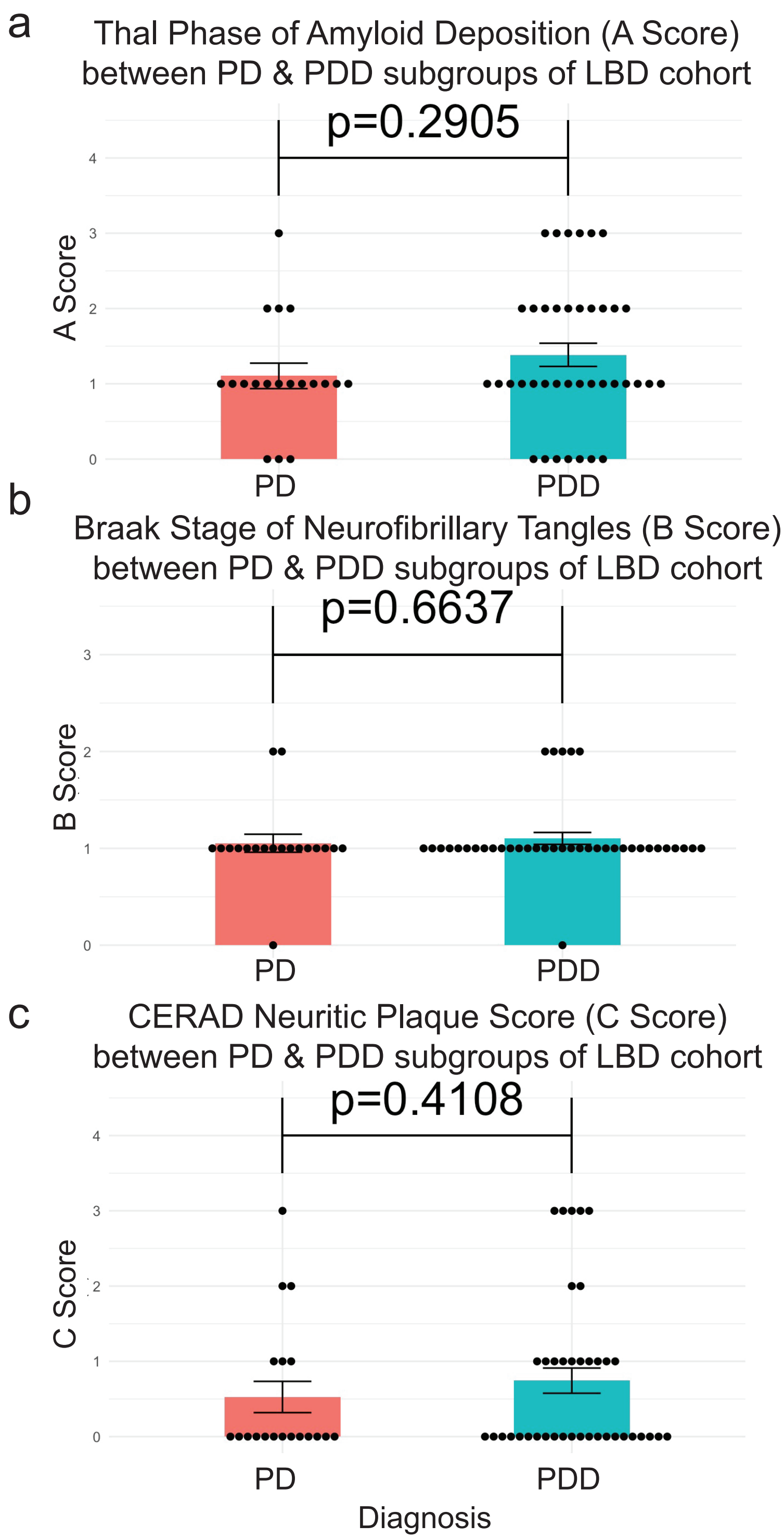

Supplemental Figure 3 PD and PDD patients exhibit similar ABC scores. Bar charts of **a)** Amyloid Thal Phase (A Score) **b)** Braak Stage of Neurofibrillary tangles (B score), and **c)** CERAD Neuritic Plaque Score (C score) of PD and PDD patients in the LBD cohort. PD n = 19, PDD n = 39. Two tailed student T-test between patient groups. All Data are mean ± Standard Error Mean. p-values indicated on charts.

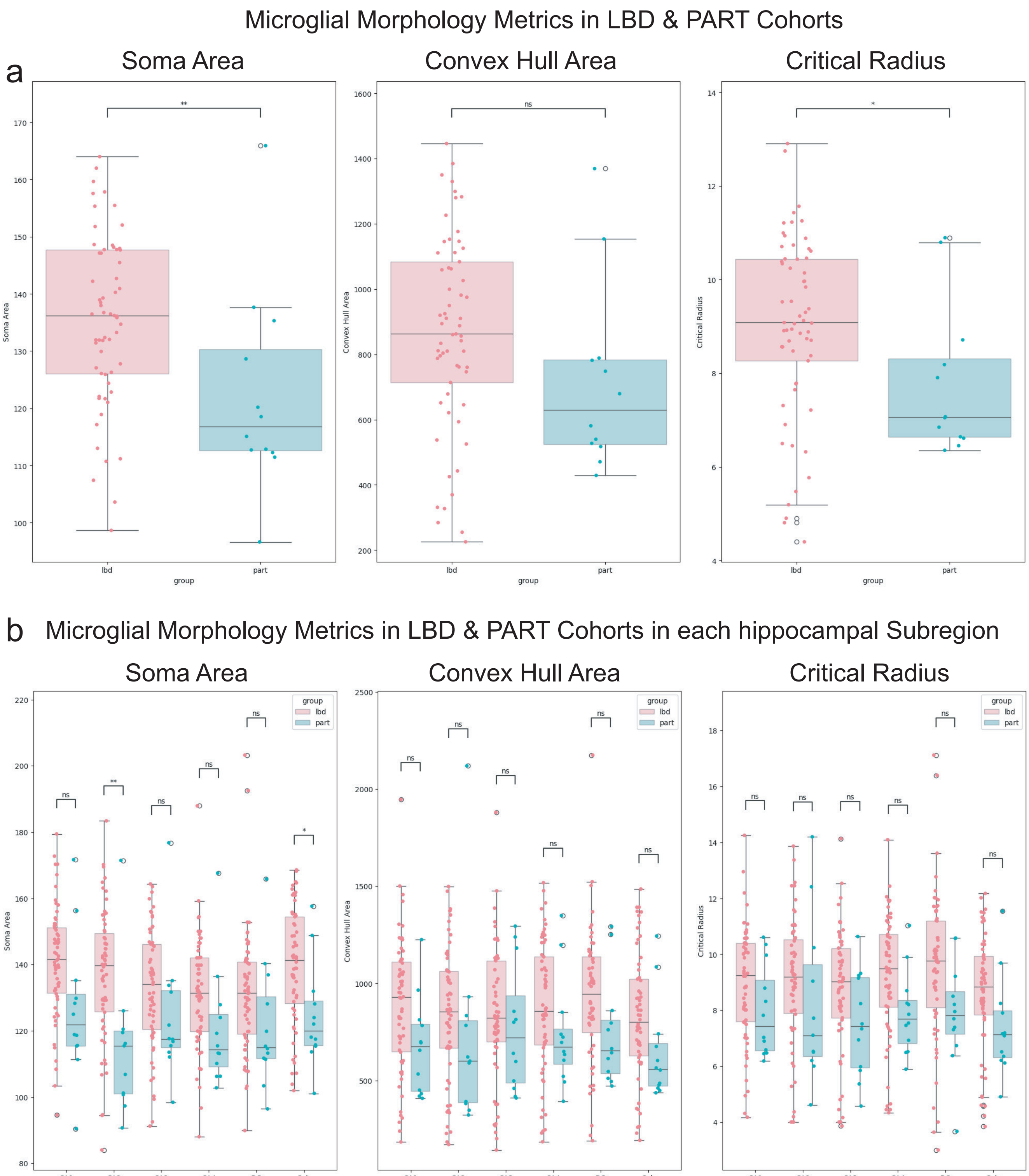

Supplemental Figure 4 Microglial morphologies between LBD and PART cohorts. a) Average microglial morphology measurements of all measured microglia throughout all hippocampal subfields between LBD and PART cohorts. **b)** Average microglial morphology measurements between LBD and PART cohorts split by hippocampal subfield. Each point is a patient sample. Y-axis units are in pixels. Statistical analysis done by t-test with bonferroni correction. ** $p<0.01$, * $p<0.05$, ns = no significant difference.

Stain	Iba1			HLA-DR			CD68		
**Predictors**	**β**	**SE**	**p**	**β**	**SE**	**p**	**β**	**SE**	**p**
Region*									
Subiculum	-0.22	0.08	**<0.01**	-0.99	0.08	**<0.001**	-0.62	0.08	**<0.001**
CA1	-0.14	0.08	0.07	-0.73	0.08	**<0.001**	-0.53	0.08	**<0.001**
CA3	-0.09	0.08	0.27	-0.24	0.08	**<0.01**	-0.10	0.08	0.19
CA4	-0.32	0.08	**<0.001**	-0.62	0.08	**<0.001**	-0.29	0.08	**<0.001**
DG	-0.18	0.08	**<0.05**	-0.72	0.08	**<0.001**	-0.66	0.08	**<0.001**
Clinical Subgroup**									
PD	0.77	0.96	0.43	-0.66	0.97	0.50	-0.34	0.59	0.57
PDD	0.11	0.92	0.91	-0.84	0.93	0.37	-0.42	0.57	0.46
Sex [Male]	-0.29	0.41	0.49	0.82	0.41	0.05	0.79	0.25	**<0.01**
Race***									
Unknown	2.14	1.82	0.19	-0.10	1.85	0.95	2.26	1.13	0.05
White	0.79	0.83	0.20	0.19	0.84	0.82	1.22	0.51	**<0.05**
Secondary Neuropathologic Diagnosis****									
AGD	-1.61	1.39	0.26	-1.61	1.40	0.26	-0.13	0.86	0.88
CVD	-0.53	0.72	0.46	-0.50	0.73	0.50	-0.72	0.45	0.11
CTE	1.32	1.34	0.33	1.39	1.35	0.31	0.13	0.83	0.88
LATE	-1.01	0.79	0.21	-0.08	0.79	0.92	0.12	0.48	0.80
PART	-1.41	0.75	0.07	0.29	0.75	0.69	-0.06	0.46	0.90
TU	-0.90	2.12	0.67	0.99	2.15	0.65	-0.77	1.32	0.56
Thal stage of amyloid deposition (A Score)	-0.08	0.34	0.81	-0.19	0.34	0.58	-0.23	0.21	0.29
Braak Stage of neurofibrillary tangles (B Score)	0.68	0.50	0.18	0.42	0.50	0.41	-0.01	0.31	0.98
CERAD neuritic plaque score (C Score)	0.16	0.25	0.52	-0.29	0.25	0.24	-0.08	0.15	0.61
Post mortem Interval	0.05	0.02	**<0.05**	0.03	0.02	0.09	0.02	0.01	0.17
LBD Stage	0.52	0.47	0.27	-0.61	0.48	0.21	-0.54	0.29	0.07
Age at Death	-0.08	0.03	**<0.01**	0.02	0.03	0.53	0.01	0.02	0.56
(Intercept)	4.57	2.49	0.07	-1.01	2.51	0.69	-2.36	1.54	0.13

Supplemental Table 1 Linear mixed effect model for distribution of Iba1, HLA-DR and CD68 in LBD cohort hippocampus. *CA2 is the reference category. ** DLB is the reference category. ***None-white patients is the reference category. **** Low probability of Alzheimer's disease is the reference category. All coefficients for the variables reported in comparison to the reference category.

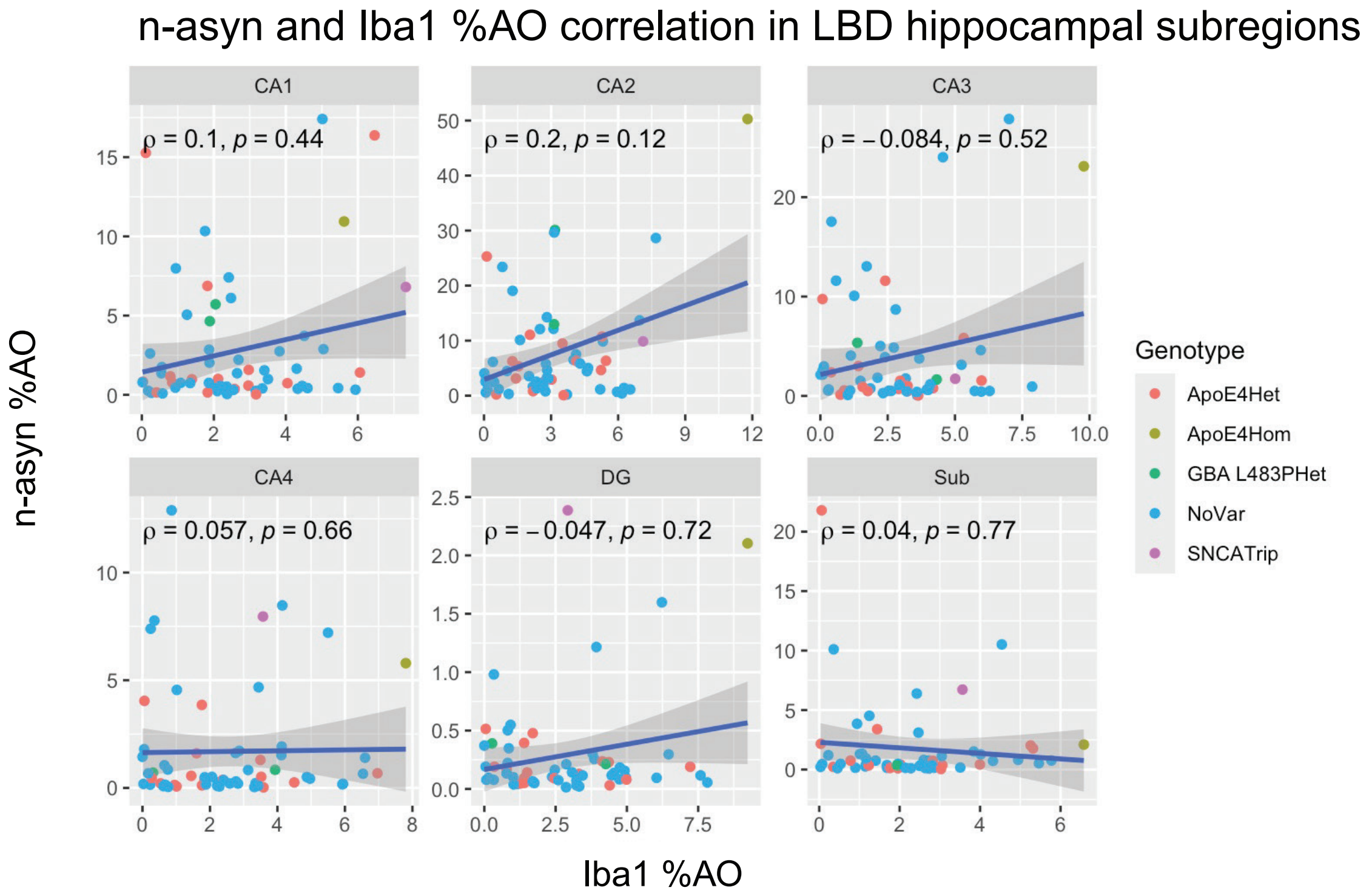

Supplemental Figure 5 Correlation of Iba1 and n-asyn %AO in hippocampal subfields of LBD cohort. Spearman rank correlation of n-asyn %AO in indicated subfields with Iba1 %AO. Spearman rank correlation coefficient and p-values provided on charts. Blue line is best fit line, and dark grey is the confidence interval. ApoE4Het = ApoE4 Heterozygous, ApoE4Hom = ApoE4 Homozygous, GBA L483PHet = GBA Leucine 483 Proline Heterozygous, NoVar = No known genetic variant, SNCATrip = SNCA gene triplication.

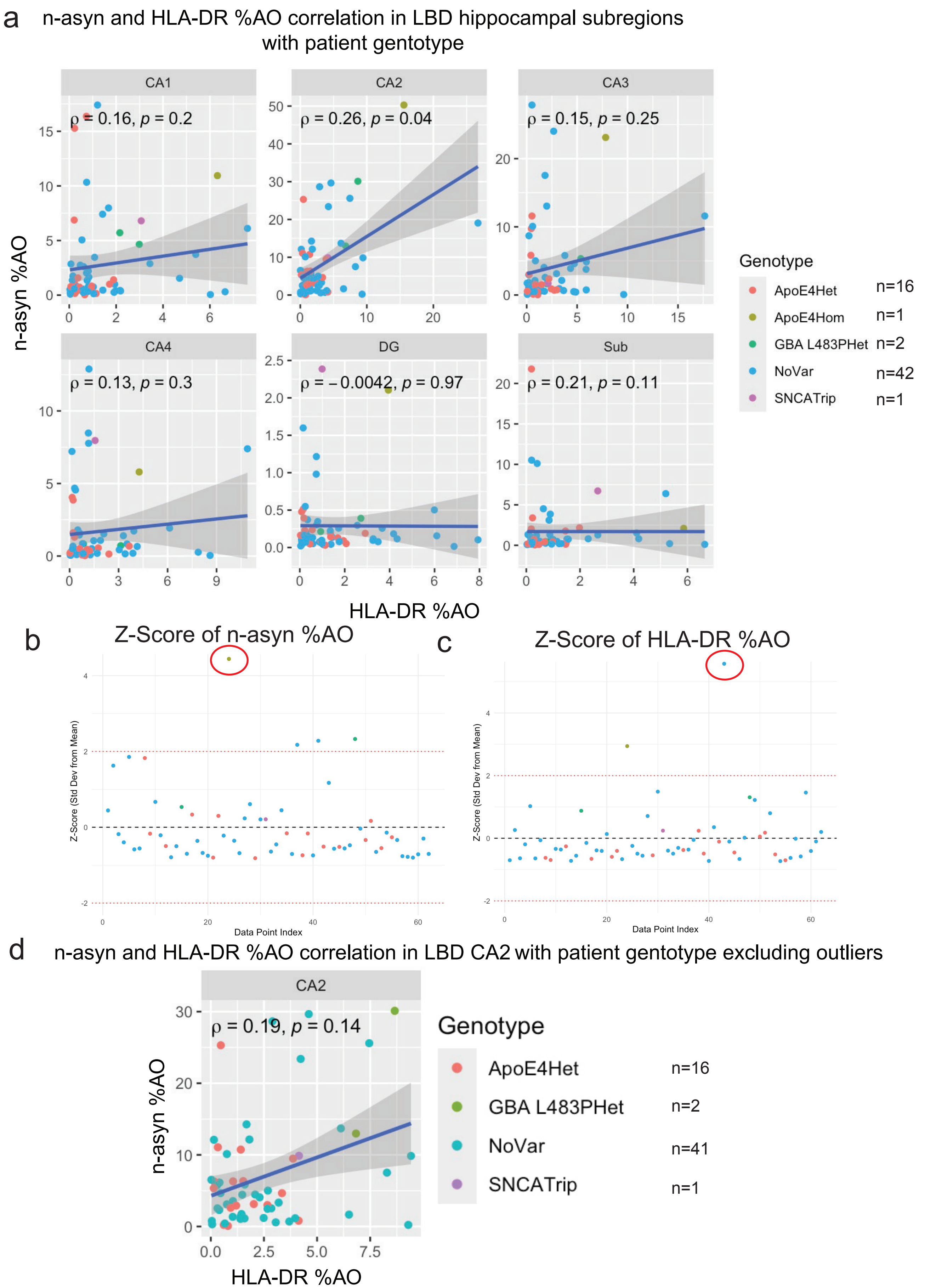

Supplemental Figure 6 Correlation of HLA-DR and n-asyn %AO in hippocampal subfields of LBD cohort with known patient genotypes. a) Spearman rank correlation of n-asyn %AO in indicated subfields with HLA-DR %AO. **b)** Z Scores of n-asyn %AO of LBD cohort in CA2 subfield. **c)** Z scores of HLA-DR %AO of LBD cohort in CA2 subfield.Std Dev = Standard Deviation. Red dotted line indicates +/- 2 Std Dev. Outliers above 3 Std Dev outlined with red circle. Legend in Panel a applied to Panels b & c. **d)** Spearman rank correlation of n-asyn %AO in the CA2 subfield with HLA-DR %AO and outliers exluded. ApoE4Het = ApoE4 Heterozygous, ApoE4Hom = ApoE4 Homozygous, GBA L483PHet = GBA Leucine 483 Proline Heterozygous, NoVar = No known genetic variant, SNCATrip = SNCA gene triplication. Correlation coefficient and p-values provided on charts. Blue line is best fit line, and dark grey is the confidence interval.

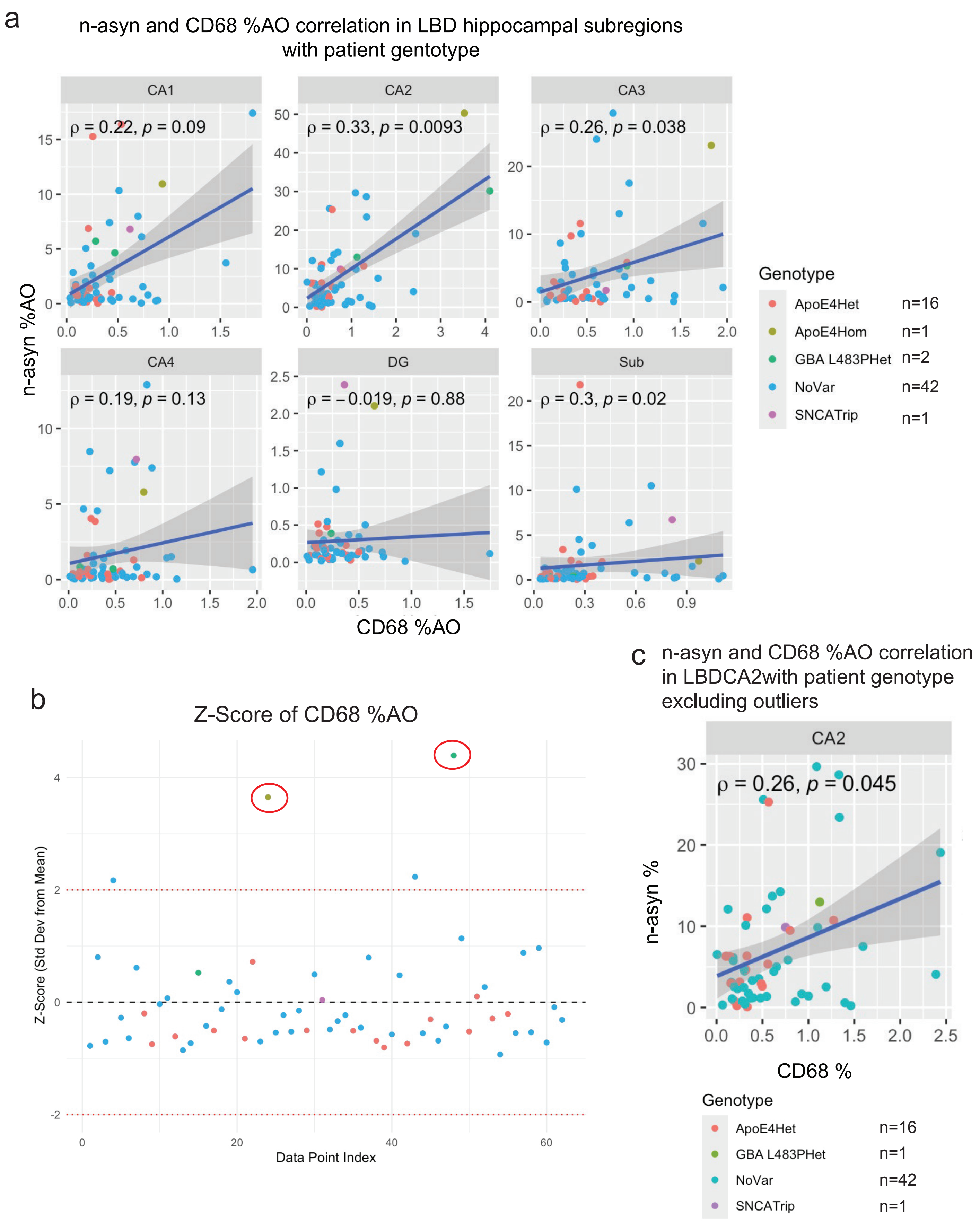

Supplemental Figure 7 Correlation of CD68 and n-asyn %AO in hippocampal subfields of LBD cohort with known patient genotypes. a) Spearman rank correlation of n-asyn %AO in indicated subfields with CD68 %AO. **b)** Z scores of CD68 %AO of LBD cohort in CA2 subfield.Std Dev = Standard Deviation. Red dotted line indicates +/- 2 Std Dev. Outliers above 3 Std Dev outlined with red circle. **c)** Spearman rank correlation of n-asyn %AO in the CA2 subfield with CD68 %AO and outliers exluded. ApoE4Het = ApoE4 Heterozygous, ApoE4Hom = ApoE4 Homozygous, GBA L483PHet = GBA Leucine 483 Proline Heterozygous, NoVar = No known genetic variant, SNCATrip = SNCA gene triplication. Legend in Panel a applied to Panel b. Correlation coefficient and p-values provided on charts. Blue line is best fit line, and dark grey is the confidence interval.

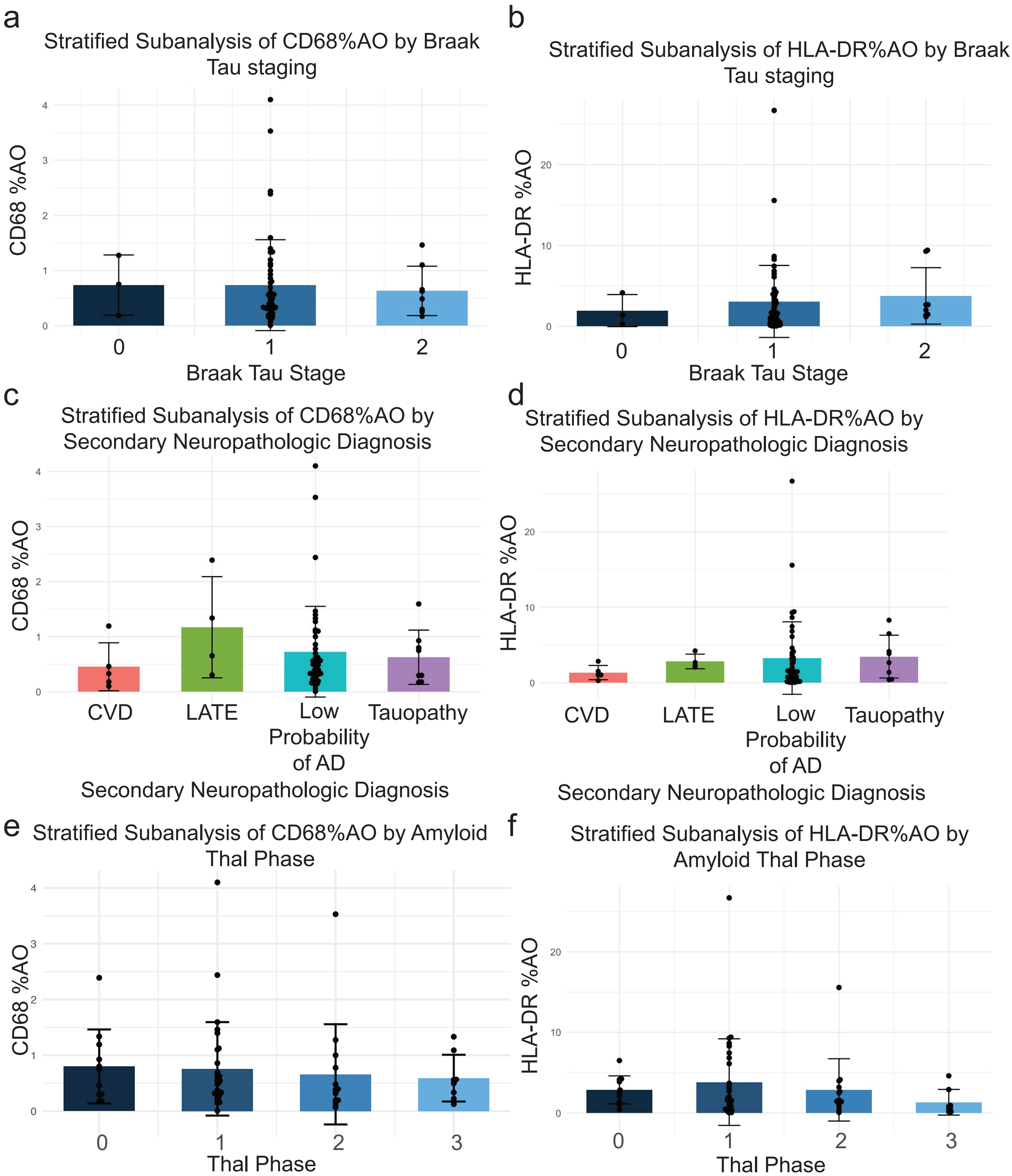

Supplemental Figure 8 Stratified Subanalysis of CD68 and HLA-DR %AO by Braak Tau stage, secondary neuropathologic diagnosis, and Amyloid Thal Phase. a) Bar chart of CD68 %AO statified by Braak Tau stage **b)** Bar chart of HLA-DR %AO statified by Braak Tau stage and secondary neuropathologic diagnosis. **c)** Bar chart of CD68 %AO stratified by secondary neuropathologic diagnosis. **d)** Bar chart of HLA-DR %AO stratified by secondary neuropathologic diagnosis. **e)** Bar chart of CD68 %AO statified by Amyloid Thal Phase. **f)** Bar chart of HLA-DR %AO statified by Amyloid Thal Phase. Each point is an individual patient. Tauopathy group is made up of PART, AGD, CTE, and Tauopathy Unclassifiable. All Data are mean ± SD. Statistical analysis done by Kruskal Wallis test with Dunn's Multiple Comparison Test.

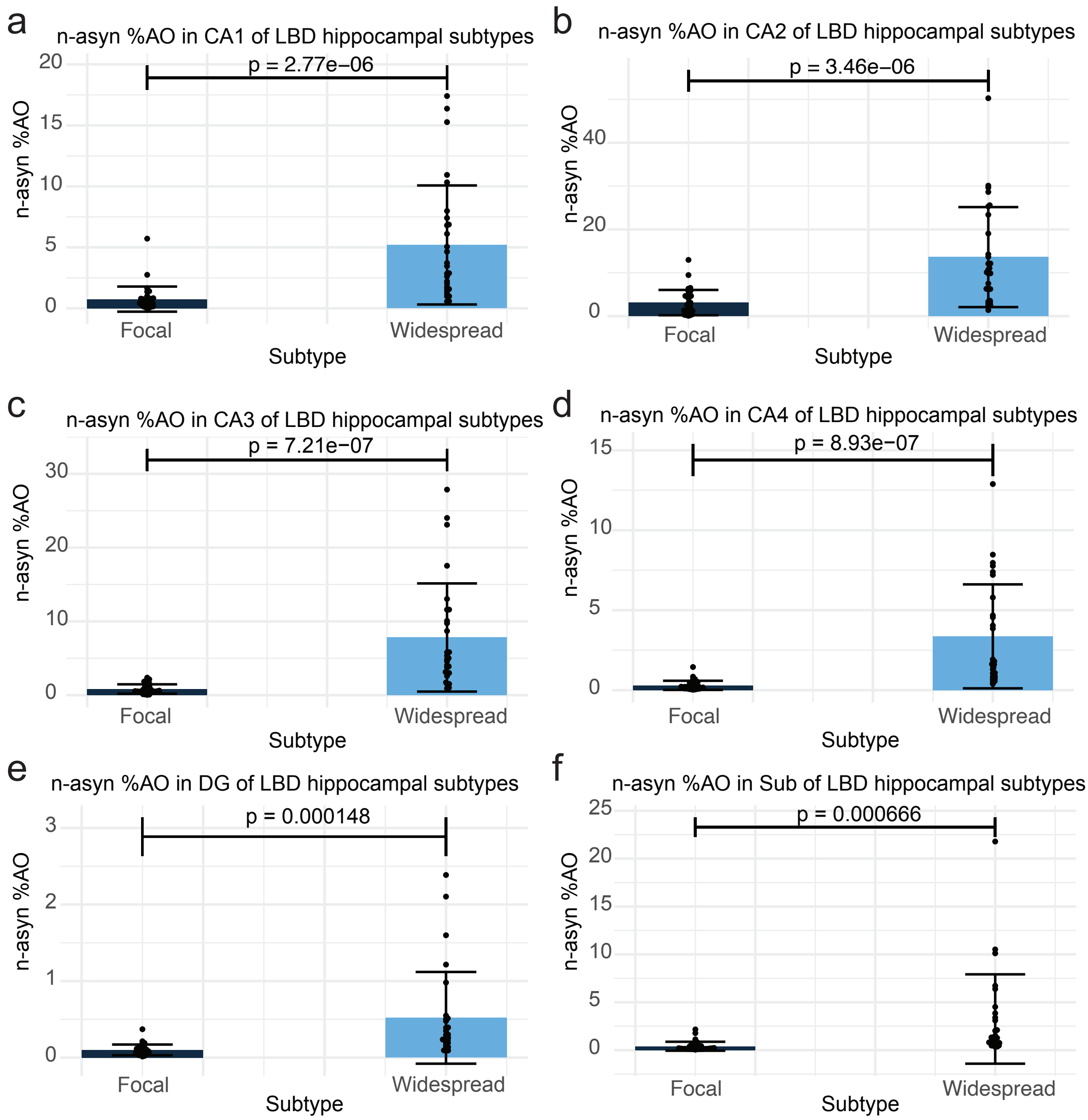

Supplemental Figure 9 Widespread subtype patients exhibit increased n-asyn %AO in all hippocampal subfields.
Widespread subtype n = 28 in indicated hippocampal subfields. Two tailed student T-test of n-asyn %AO between patient groups. All Data are mean ±SD. p-values indicated on charts.

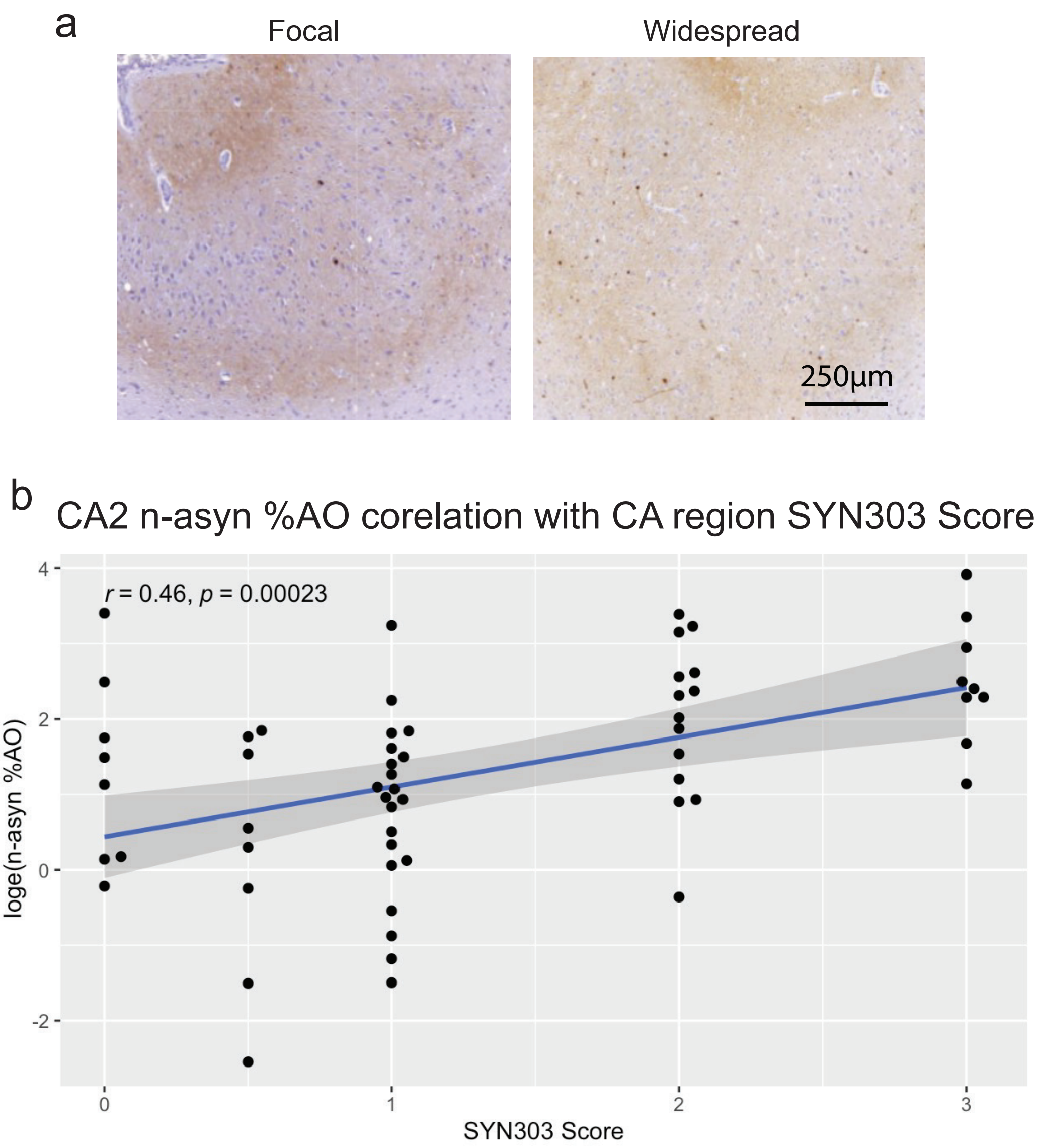

Supplemental Figure 10 Greather n-asyn burden measured by MJFR13 correlates with increased n-asyn burden measured by SYN303 Ordinal Rating. a) Representative images of SYN303 staining from the hippocampal CA2-3 region of indicated patient subtypes. Scale bar indicated on image. **b)** Pearson correlation of natural log transformed n-asyn %AO in the CA2 subregion of the LBD cohort with SYN303 rating score in the CA region of the hippocampus. n=60. Correlation coefficient and p-values provided on charts. Blue line is best fit line, and dark grey is the confidence interval.

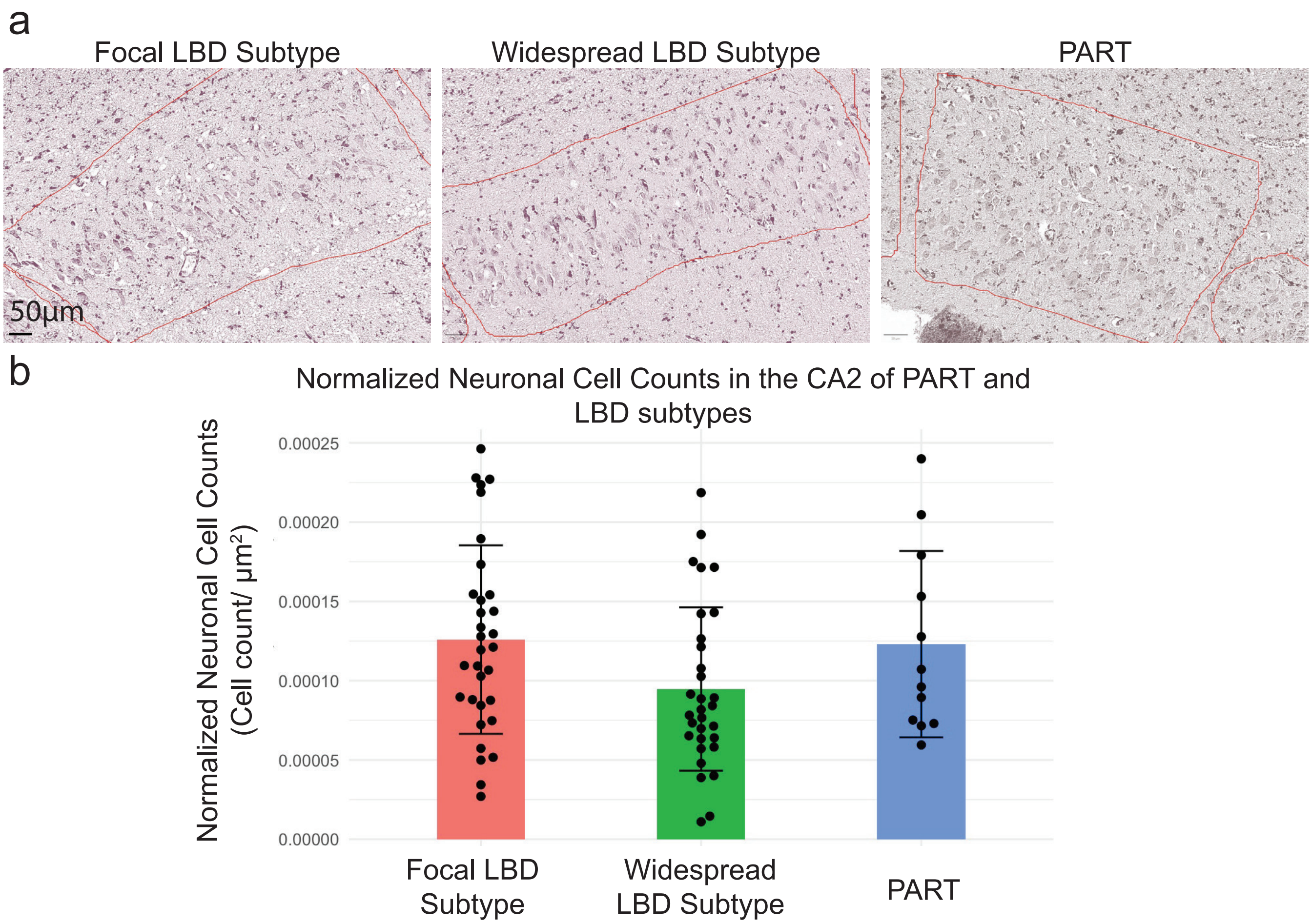

Supplemental Figure 11 Normalized Cell Counts by area in patient subgroups. a) Representative images of the Hematoxylin channel from CA2 subfield from CD68 stained slides for Focal & Widespread LBD subtypes and PART cohort. Red outline is the subfield annotation. Scale bar indicated in image. **b)** Bar chart of cell counts per µm2 in indicated groups. Each point is an individual patient. All Data are mean ± SD. Statistical analysis done by one way anova with Tukey's post hoc analysis.

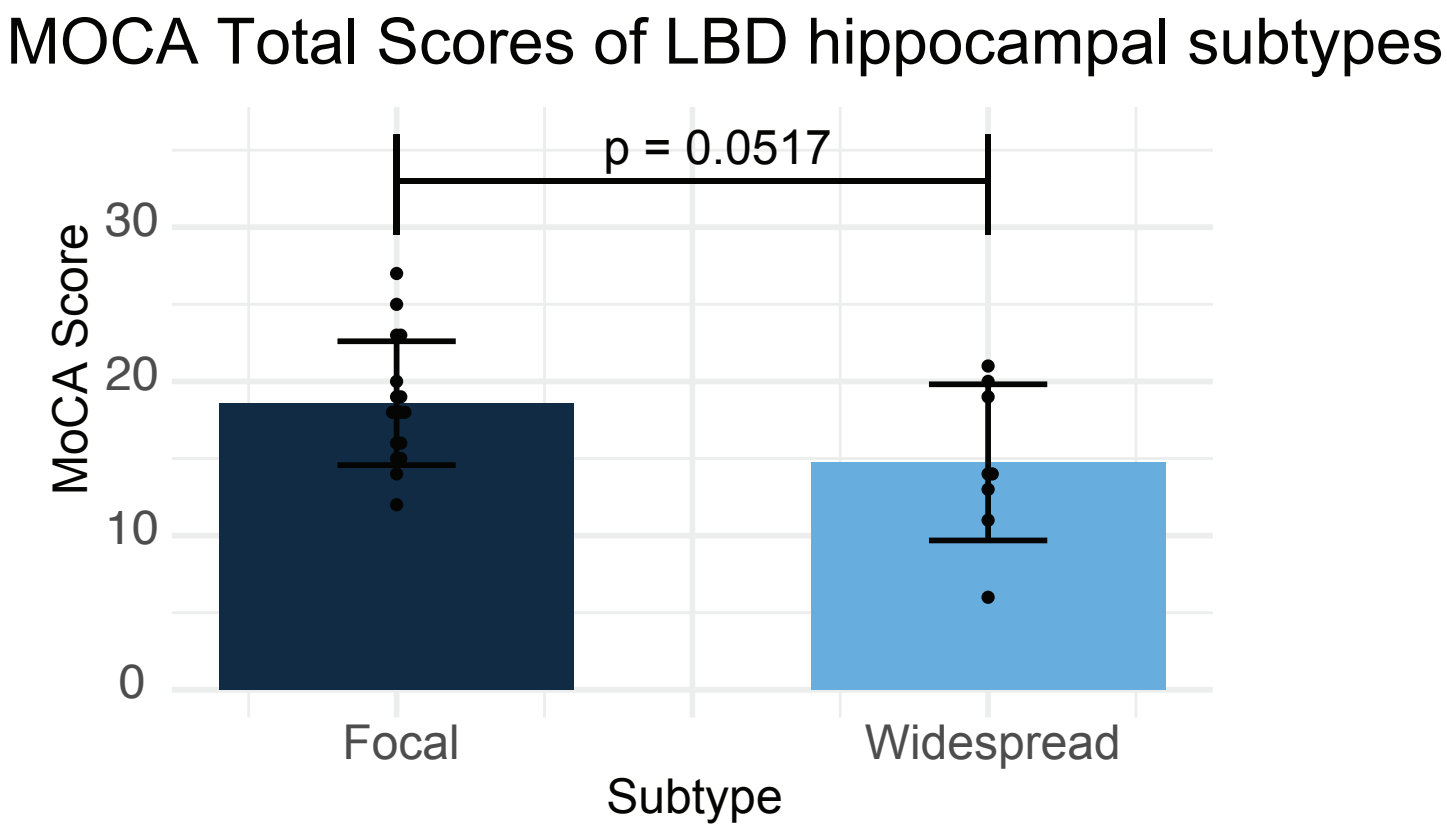

Supplemental Figure 12 Comparison of MOCA scores between Focal and Widespread subtype patients. Bar chart of MOCA scores in Focal subtype n = 17 Widespread subtype n = 8. Two tailed student T-test of MOCA Total Scores between patient groups. All Data are mean ± SD. p-value indicated on charts.

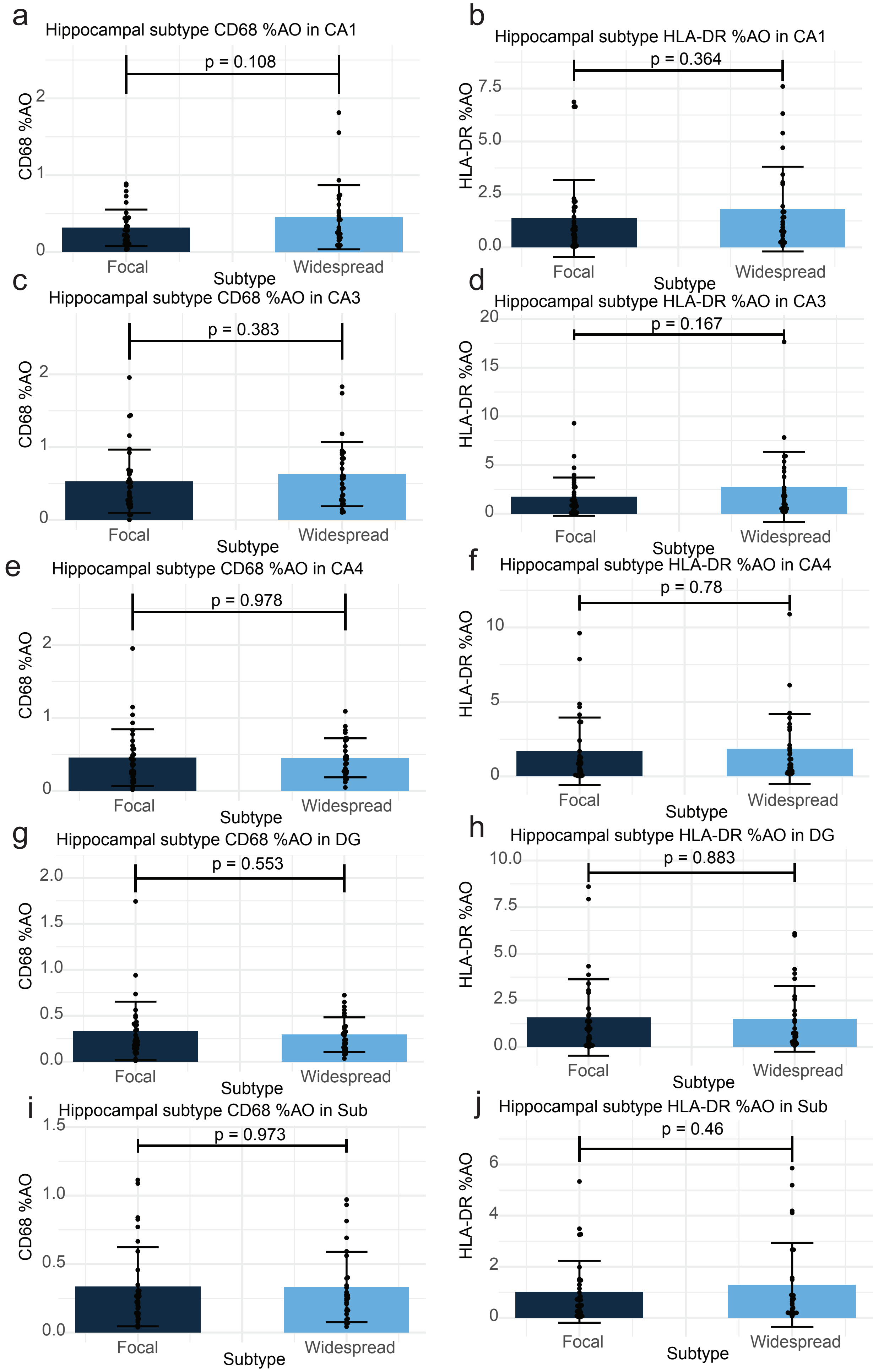

Supplemental Figure 13 There is no difference in microglial reactivity in any hippocampal subregion outside the CA2 between Focal and Widespread subtype patient groups. (a-j) HLA-DR and CD68 %AO within each hippocampal subregion outside the CA2, indicated on charts, in Focal and Widespread subtype patient groups. in Focal subtype n = 34 and Widespread subtype n = 28. Two tailed student T-test. All Data are mean ± SD. p-value indicated on charts.

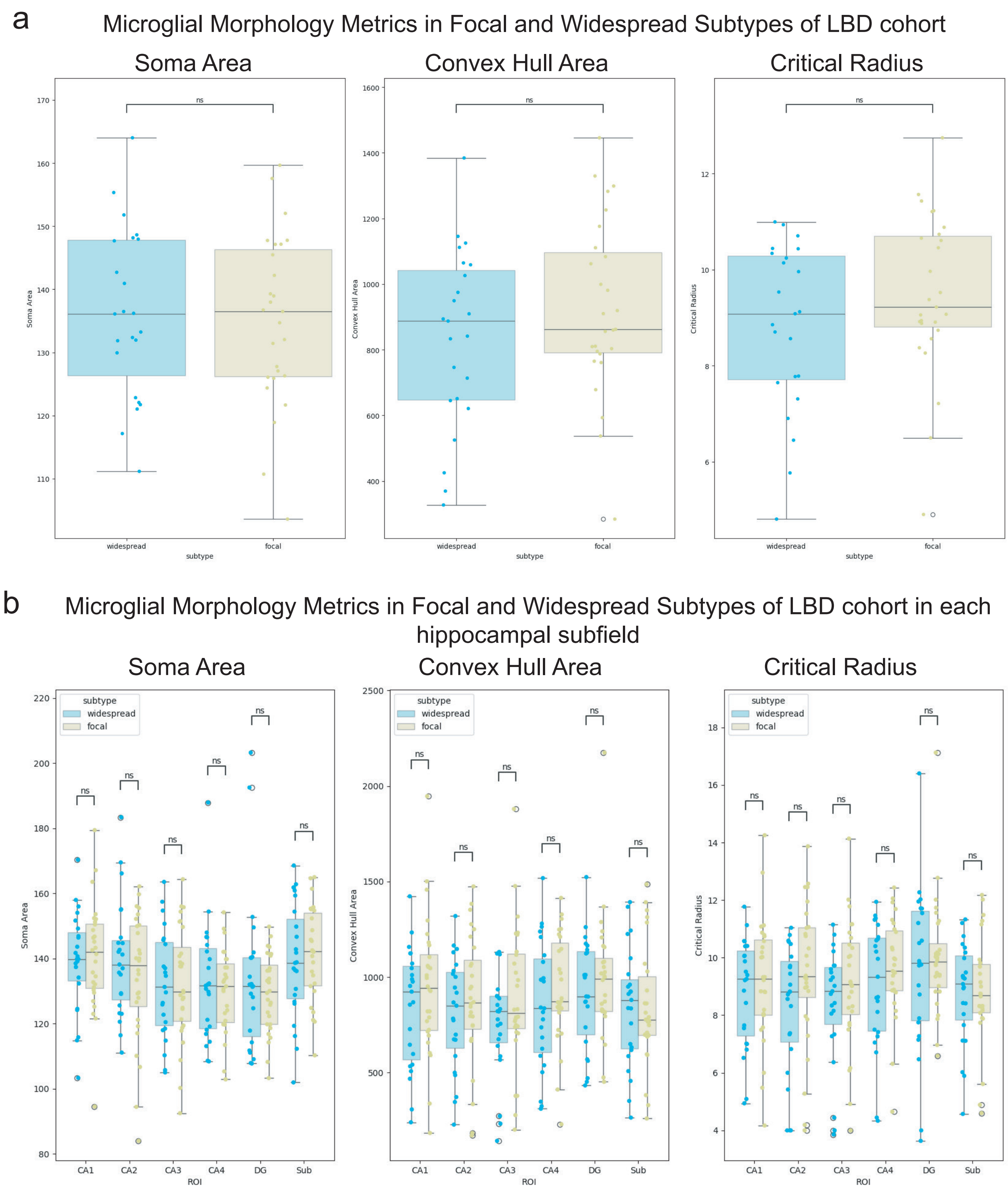

Supplemental Figure 14 Microglial morphologies between Focal and Widespread subtypes of LBD cohort. a) Average microglial morphology measurements of all measured microglia throughout all hippocampal subfields between Focal and Widspread LBD subtypes. **b)** Average microglial morphology measurements between Focal and Widspread LBD subtypes split by hippocampal subfield. Y-axis units are in pixels. Each point is a patient sample. Statistical analysis done by t-test with bonferroni correction. ns = no significant difference.

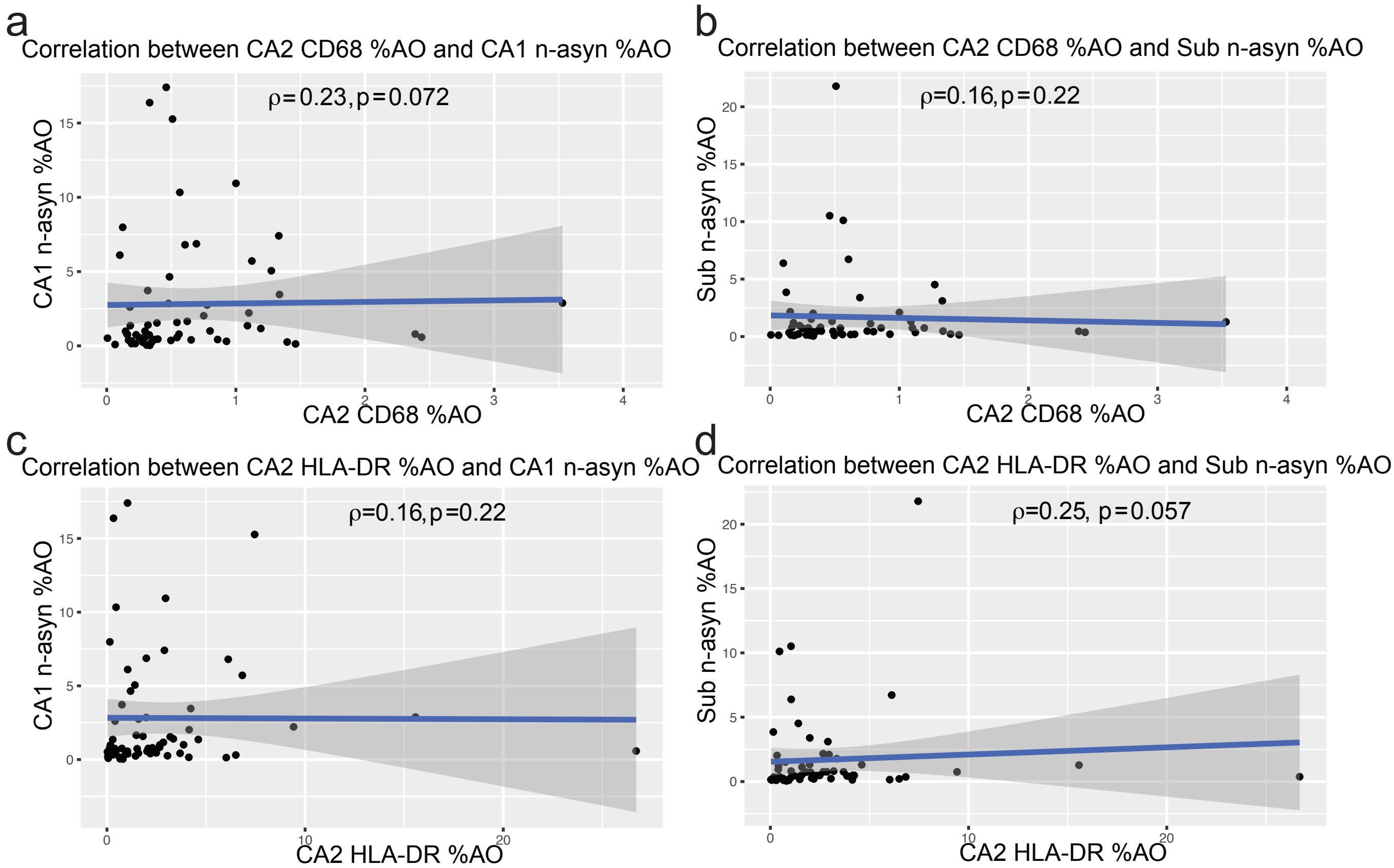

Supplemental Figure 15 Between region correlations of focal CA2 microglial reactivity and n-asyn %AO in anterograde portions of the intrahippocampal circuit. (A) Spearman rank correlations of CA2 CD68 %AO with n-asyn %AO in CA1 and (B) Sub. (C), Spearman rank correlations of CA2 HLA-DR %AO with n-asyn %AO in CA1 and (D) Sub. Spearman rank correlation coefficient and p-values provided on charts. Blue line is best fit line, and dark grey is confidence interval.
